# Energy Metabolism in H460 Lung Cancer Cells: Effects of Histone Deacetylase Inhibitors

**DOI:** 10.1371/journal.pone.0022264

**Published:** 2011-07-18

**Authors:** Nívea Dias Amoêdo, Mariana Figueiredo Rodrigues, Paula Pezzuto, Antonio Galina, Rodrigo Madeiro da Costa, Fábio Ceneviva Lacerda de Almeida, Tatiana El-Bacha, Franklin David Rumjanek

**Affiliations:** Instituto de Bioquímica Médica, Universidade Federal do Rio de Janeiro, Cidade Universitária, Rio de Janeiro, Brazil; Instituto de Química - Universidade de São Paulo, Brazil

## Abstract

**Background:**

Tumor cells are characterized by accelerated growth usually accompanied by up-regulated pathways that ultimately increase the rate of ATP production. These cells can suffer metabolic reprogramming, resulting in distinct bioenergetic phenotypes, generally enhancing glycolysis channeled to lactate production. In the present work we showed metabolic reprogramming by means of inhibitors of histone deacetylase (HDACis), sodium butyrate and trichostatin. This treatment was able to shift energy metabolism by activating mitochondrial systems such as the respiratory chain and oxidative phosphorylation that were largely repressed in the untreated controls.

**Methodology/Principal Findings:**

Various cellular and biochemical parameters were evaluated in lung cancer H460 cells treated with the histone deacetylase inhibitors (HDACis), sodium butyrate (NaB) and trichostatin A (TSA). NaB and TSA reduced glycolytic flux, assayed by lactate release by H460 cells in a concentration dependent manner. NaB inhibited the expression of glucose transporter type 1 (GLUT 1), but substantially increased mitochondria bound hexokinase (HK) activity. NaB induced increase in HK activity was associated to isoform HK I and was accompanied by 1.5 fold increase in HK I mRNA expression and cognate protein biosynthesis. Lactate dehydrogenase (LDH) and pyruvate kinase (PYK) activities were unchanged by HDACis suggesting that the increase in the HK activity was not coupled to glycolytic flux. High resolution respirometry of H460 cells revealed NaB-dependent increased rates of oxygen consumption coupled to ATP synthesis. Metabolomic analysis showed that NaB altered the glycolytic metabolite profile of intact H460 cells. Concomitantly we detected an activation of the pentose phosphate pathway (PPP). The high O_2_ consumption in NaB-treated cells was shown to be unrelated to mitochondrial biogenesis since citrate synthase (CS) activity and the amount of mitochondrial DNA remained unchanged.

**Conclusion:**

NaB and TSA induced an increase in mitochondrial function and oxidative metabolism in H460 lung tumor cells concomitant with a less proliferative cellular phenotype.

## Introduction

The unchecked proliferation and invasion typical of cancer cells are processes that can only be sustained when there is sufficient energy supply, a feature that indicates the occurrence in transformed cells of distinct phenotypes that necessarily involve elements of the intermediary metabolism. In solid tumors it has been shown by Otto Warburg that cells have adapted to rely on anaerobic glycolysis as a strategy to maintain their prevailing anabolic status [1]. However, the upregulation of glycolysis exhibited by cancer cells does not necessarily imply a strict anaerobic phenotype nor a dysfunctional oxidative phosphorylation system (OXPHOS). Rather, it is believed that the normal interplay between the glycolysis in the cytosol and OXPHOS in the mitochondria becomes disturbed or reprogrammed in tumor cells. The Crabtree effect observed in cancer cells, or in rapidly proliferating cells exemplifies the intimate connection between glycolysis and the oxidative metabolism [2].

Interestingly, the anaerobic phenotype exhibited by cancer cells may in fact represent the cause rather than the consequence of the adaptive pressure. By considering that the glycolytic switch typical of cancer cells is acquired at the very onset of carcinogenesis, the idea arose that alterations in the glycolytic pathway may predispose cells to malignant transformation [3], [4]. Selective advantages for the transformed cells could result from various features. For instance, it is known that hypoxia-inducible factor-1 (HIF-1α) greatly stimulates the expression of glucose and monocarboxylate transporters, glycolytic enzymes and induces a down regulation in pyruvate dehydrogenase complex [5]. Moreover, tumor cells present the isoform of HK that binds to the mitochondrial pore forming protein voltage-dependent anion channel (VDAC). By preventing the interaction of pro-apoptotic proteins with mitochondria the bound enzyme acts essentially as an anti-apoptotic agent. Indeed, it has been shown that the release of apoptotic proteins such as cytochrome *c* depends on the integrity of the N-terminal portion of VDAC [6]. Since it was demonstrated that HK and Bcl-2 were able to confer protection against apoptosis through interaction with the VDAC 1 N-terminal region, the participation of HK II as a promoter of cell differentiation was strengthened.

Enzymes of the glycolytic and oxidative pathways are, as proteins in general, amenable to regulation of gene expression at the level of chromatin. Chromatin structures alternate between compacted and relaxed conformations which in turn depend on acetylation and deacetylation of the histone protein core. The enzymatic systems involved in these processes are histones acetyl transferases (HATs) that add acetyl groups to lysine residues and histone deacetylases (HDACs) that remove them. Compacted and relaxed chromatins have been linked to gene expression repression and activation, respectively. Although histones constitute the prime substrates for HATs and HDCAs, other non-histone proteins such as transcriptional factors-p53, pRb retinoblastoma protein and HIF-1α; chaperones (HSP90), metabolic enzymes (pyruvate kinase; acetyl-CoA synthase) and steroid receptors are also acetylated/deacetylated by these enzymes. Therefore, HATs and HDACs can affect a broad spectrum of biological processes that include growth arrest, DNA repair, cellular bioenergetics, cell death pathways (apoptosis, and autophagy), mitosis, generation of reactive oxygen species (ROS), senescence and angiogenesis[7], [8]. Because of its repressive actions, HDACs have become interesting targets for the development of drugs that could retrieve the ability of transformed cells to undergo apoptosis. Currently, several HDAC inhibitors (HDACis) obtained from natural or synthetic sources have been characterized. They are grouped into five chemical classes which include hydroxamic acid and derived compounds, benzamides, cyclic peptides, short chain fatty acids and ketones.

As mentioned above, the HDACis change several functions in normal and transformed cells which makes it difficult to pinpoint a mechanism of action to these drugs. Several reports exist showing the action of HDACi on the cell cycle and apoptosis. The present work has dissected these broad actions by focusing on the energy metabolism and demonstrating that HDACis can affect proliferation by acting on individual enzymes of the glycolytic and oxidative pathways. The information available so far studying mitochondria from rat liver treated with the short chain fatty acid derivative valproate (VPA) have shown that it inhibits fatty acid β-oxidation and in general depresses cell oxidative metabolism leading to a decrease in both, the rate of O_2_ consumption coupled to ATP synthesis and cytochrome oxidase activity [9], [10], [11]. Colorectal adenocarcinomas cells (HT29) treated with butyrate, another short chain fatty acid class HDACi, inhibited glucose uptake and oxidation, as well as ribose synthesis and increased *de novo* fatty acid synthesis along with activation of the PPP. However MIA cells, butyrate-resistant pancreatic adenocarcinoma, did not display any changes in their metabolic profile after treatment. These metabolic changes were correlated to induction of differentiation processes mediated by butyrate and consequently with its inhibitory effects on growth. Similar results were obtained with cells exposed to TSA [12], [13]. In myeloma cells, the HDACis VPA and suberoylanilide hydroxamic acid (SAHA) induced a decrease in glucose uptake, GLUT 1 expression and HK activity, leading to apoptosis in tumor cells. In addition, these inhibitors increased the amino acid catabolism [14].

The present study examined the roles of NaB and TSA on several parameters, biochemical and morphological, of the H460 cell line of lung cancer cells in order to clarify how these HDACis interferes with tumor cell homeostasis. The data showed conclusively that treatment with NaB for 24 h lead to a generally enhanced oxidative metabolism clearly suggesting that HDACis may transcend their canonical role at the chromatin level.

## Methods

### Cell Culture

H460, a human lung cancer cell (ATCC, deposited by AF Gazdar, 1982), was maintained in RPMI 1640 medium supplemented with 10% fetal bovine serum (FBS), pH 7.4 at 37°C in a humidified incubation chamber with 5% CO_2_. Cells were sub-cultured every 2 days and used on experiments when they reached 85% confluence. The H460 cells were genotyped in our laboratory using 8 loci plus amelogenin. All loci matched the ATCC DNA STR profile.

### Cell Viability and Citotoxicity Assay

For those assays the cells were grown on 24 and 96 well plates. 24 h after plating, cells were incubated with three different concentrations of NaB (1, 3 and 10 mM) and TSA (0.02, 0.2 and 1 µM) for up to 48 h. After each treatment, cell viability was accessed using MTT assay, as described previously [15], and trypan blue dye exclusion assay. Citotoxicity was assayed by lactate dehydrogenase (LDH) release after NaB treatment using CytoTox96 Non-radioactive Cytotoxicity assay kit (Promega).

### Cell Cycle Analysis

For DNA staining H460 cells were grown in 6 well plates and treated with 3 or 10 mM NaB and 0.2 µM TSA for 24 h. The cells were pelleted (1000 *x g* for 5 min. at 4°C) and ressuspended in 500 uL of PI staining solution, composed of 50 ug/mL propidium iodide (PI), 1 mg/mL RNAse and 0.2% Triton X-100. Samples were incubated on ice for 15 min. in the dark and then analyzed in FACSCalibur flow cytometer (Becton Dickinson) using CellQuest software (Becton Dickinson), respectively, for data acquisition and cell cycle distribution analysis.

### Kinetics of Lactate Release in the Culture media

After treatment with different concentrations of NaB and TSA for 24 h, the culture medium was replaced by fresh RPMI 1640 medium without phenol red and FBS. At the indicated times (0, 15, 30, 40, 50 and 60 min.), aliquots from culture medium were collected to evaluate lactate release through this enzymatic assay: lactate measurement was performed in a hydrazine/glycine buffer (pH 9.2), containing 5 mg/mL β-NAD^+^ and 15 units/mL lactate dehydrogenase. The absorbance due to formation of NADH was monitored in a microplate reader (SpectraMax M5, Molecular Devices) at 340 nm and was correlated with the presence of lactate on samples from a standard curve [16].

### Preparation of Mitochondrial and Cytosolic Protein Fractions

H460 cell pellet was mixed to a lysis buffer containing 10 mM Tris-HCl pH 7.4, 0.25 M sucrose, 20 mM NaF, 1 mM DTT, 5 mM EDTA, 1 mM PMSF and protease inhibitors cocktail (chymostatin, leupeptin, antipain, pepstatin A – Sigma-Aldrich). The cell suspension was transferred to homogenizers (Wheaton Potter-Elvehjem) for cell lysis. The suspension was centrifuged at 100 *x g* for 5 min. at 4°C, the pellet with debris was discarded and the resulting supernatant was centrifuged at 10000 *x g* for 15 min. at 4°C. Then the supernatant (cytosolic fraction) was used for measurement of the recovered activities of HK, PYK, LDH and glucose-6-phosphate dehydrogenase (G6PDH) and the pellet (mitochondrial fraction) was used for measurement of the recovered activities of mt-HK and CS. These extracts were used for western blotting and enzymatic activity assays. Protein concentration was performed using the Bradford method.

### Enzymatic Activity Assays

Enzymatic activities were measured using the following reaction media: (a) for HK, 20 mM Tris-HCl pH 7.4, 10 mM MgCl_2_, 1 mM β-NAD^+^, 1 unit/mL G6PDH (*Leuconostoc mesenteroides*), 0.1% Triton X-100, 2 mM ATP and 5 mM glucose. (b) For PYK, 50 mM Tris-HCl pH 7.4, 5 mM MgCl_2_, 50 mM KCl, 0.2 mM β-NADH, 2.5 mM ADP, 0.1% Triton X-100, 5 mM phosphoenolpyruvate, 0.5 units/mL lactate dehydrogenase. (c) For LDH, 50 mM Tris-HCl pH 7.4, 1 mM EDTA, 0.2 mM β-NADH, 0.1% Triton X-100, 1 mM pyruvate. (d) For G6PDH, 50 mM Tris-HCl pH 7.4, 5 mM MgCl_2_, 0.1% Triton X-100, 2 mM glucose-6-phosphate and 0.2 mM β-NADH. The reactions were started by the addition of 20–140 µg protein for HK and 10 µg protein for PYK, LDH and G6PDH and were carried out at 37°C for 5 min. After incubation, the samples were immediately boiled and placed in ice. Absorbance was measured at 340 nm and the value used to calculate the specific activity of the enzymes defined as the amount of substrate formed per milligram of protein per minute. (e) For CS, 50 mM Tris-HCl pH 8.1, 0.3 mM acetyl-CoA, 0.1% Triton X-100, 0.1 mM DTNB, 0.5 mM oxaloacetate. The reaction was started by the addition of 10 µg protein and carried out at 30°C for 5 min. Absorbance was measured at 412 nm. (f) For succinate dehydrogenase (SDH), 50 mM phosphate buffer (pH 7.4), 0.1% Triton X-100, 0.8 mM KCN, 0.06 mM 2,6-dichlorophenolindophenol (DCIPIP), 1.1 mM phenazine (PMS) and 100 µg protein The reaction was carried out at 25°C for 7 min and was stopped with 10 mM malonate. The reduction of DCPIP was monitored at 600 nm.

### Western Blotting

25 µg of protein extracts were separated by standard SDS-PAGE and transferred to nitrocelulose membranes by electroblotting in buffer composed of 39 mM glycin, 48 mM Tris-base, 0.037% SDS and 20% methanol. Western blotting was performed using primary antibodies diluted in TBS, 0.1% Tween 20 and 5% BSA. Primary antibodies against HK I, HKII and VDAC 2 (abcam) were used.

### Oxygen Consumption of Intact and Digitonin-Permeabilized H460 cells

O_2_ consumption rates were measured polarographically using high-resolution respirometry (Oroboros Oxygraph-O2K). After the treatment period, medium was removed and cells were either suspended in RPMI (11.1 mM glucose and 2 mM glutamine) or glucose free DMEM for measurements of O_2_ consumption of intact cells, or in the respiration medium pH 7.0 (0.25 M mannitol, 10 mM MgCl_2_, 10 mM KH_2_PO_4_, 10 mM HEPES, 0.08 mM EDTA, 1 mM EGTA and 0.1% fatty acid free BSA) for evaluation of respiratory complexes of permeabilized cells. Routine consumption, oligomycin-independent respiration (proton leak) and FCCP-stimulated respiration (maximum respiration) were measured in intact H460 cells as described previously [17]. NaB effects on respiratory complexes of 0.003% digitonin-permeabilized H460 cells were performed following the addition of different substrates/modulators such as 10 mM pyruvate + 10 mM malate (complex I-linked substrates), 10 mM succinate (complex II-linked substrate), 0.5 µM rotenone, 100 µM ADP. When assaying state 3 induced by 2-deoxyglucose (2-DOG), 10 mM of 2-DOG was added. DatLab software (Oroboros Instruments, Innsbruck, Austria) was used for data acquisition and analysis.

### RNA Extraction and cDNA Synthesis

Total RNA was isolated from H460 cells using TRIzol reagent (Invitrogen) according to the manufacturer's instructions. Total RNA was quantified spectrophotometrically and 1 µg were treated with 1 U of DNAse RNAse-free for 30 min. at 37°C. Reactions were stopped by adding 1 µL of 20 mM EDTA and heating for 10 min. at 65°C. cDNA synthesis was performed using the DNAse treated RNA according to High Capacity cDNA Reverse Transcription Kit from Applied Biosystems.

### Real Time PCR

Gene expression analysis was performed using 7500 Real Time PCR (Applied Biosystems) and power SYBR-GREEN PCR master MIX (Applied Biosystems). For this test primer pairs were synthesized based on GenBank sequences of mRNA. The sequences of the primers are presented in Table S1. The comparative Ct method was used to compare changes in gene expression levels [18]. Actin was used as an endogenous control.

### Electron Microscopy

Cells were washed once in warm PBS and fixed in a solution pH 7.2 containing 2.5% glutaraldehyde, 0.1 M sodium cacodylate buffer, post- fixed with 1% OsO_4_ (osmium tetroxide) in 0.1 M sodium cacodylate buffer. Afterwards, cells were washed with PBS, dehydrated with acetone and embedded in Epon. Ultra-thin sections (70 nm) were stained with uranyl acetate and lead citrate, and observed in a Zeiss 900 electron microscope. For mitochondria morphometry, twenty electron micrographs were taken from each sample and analyzed for morphology. Measurements of each mitochondrion profiles area in ultrathin sections were made using Image J (NIH) software.

### Nuclear Magnetic Ressonance (NMR) for Metabolomic Analysis

Metabolomic screening of H460 cells was performed as described in [19] with few modifications. Briefly, culture media of non-treated and NaB-treated H460 cells were replaced by glucose-free DMEM supplemented with D-[U-^13^C] 5 mM glucose and cells were incubated for 1 h. Following incubation, the medium was removed and approximately 3×10^7^ cells were suspended in glucose-free DMEM containing 10% deuterium oxide. One-dimensional ^13^C spectra of intact H460 cells metabolites were obtained at 25°C. Spectra of H460 cells metabolites were acquired with a Brucker DRX 400 MHz using a triple resonance probe (TXI). Spectra processing and analysis were performed using Topspin 2.0 and metabolite assignment was done by chemical shift comparison of known metabolites deposited in the Human Metabolome Database v 1.0.

### Statistical Analysis

Statistical analysis was performed using Graph Pad Prism 5. The results were expressed as means ± S.E.M for *n* independent experiments. Statistical significance was determined by student's *t* test, *one-way* ANOVA and *two-way* ANOVA.

## Results

### Sodium butyrate and trichostatin A inhibited the proliferation of H460 cells and induced morphological changes compatible with a differentiation process

**Figure 1:**
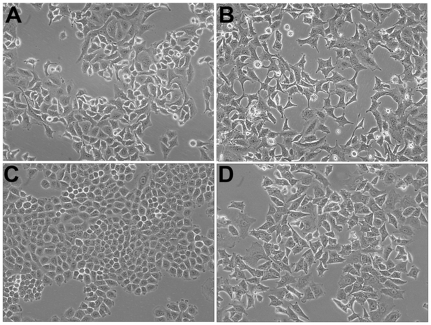
Sodium butyrate induces morphological changes and cell cycle arrest in H460 cells. The cells were incubated at 37°C humidified incubator containing 5% CO_2_ and photographed under bright field using the documentation system of inverted microscope Nikon TS100. (A) A549 cells non-treated. (B) A549 cells treated with 10 mM NaB for 24 h. (C) H460 cells. (D) H460 cells treated with 10 mM NaB for 24 h.

Initially, we performed assays to evaluate NaB and TSA effects on cell viability to determine the best experimental conditions to study HDACis effects on energy metabolism without interferences caused by cytotoxicity. As observed by phase contrast microscopy, H460 cells treated with NaB exhibited discrete differences in comparison to control cells. The morphology observed was compatible with that of differentiated cells, suggesting that NaB may have counteracted regulatory pathways that in tumor cells would lead to dedifferentiation. The results in Figure 1 show further that treatment with 10 mM NaB produced cells that were less confluent and are slightly more elongated than the untreated ones (Figure 1C and D). Interestingly, the morphology of H460 NaB treated cells resembled A549 cells, a more differentiated lung cancer cell that does not show morphological changes upon NaB treatment (Figure 1A and B). These alterations in cell shape were possibly related to cytoskeleton reorganization, since treatment of cells with NaB produced a marked redistribution of F-actin as revealed by staining with rhodamine- labeled phaloidin (Figure S1; Method S2). Based on this observation the question whether 10 mM NaB could have had cytotoxic effects on the cultures was raised. Experiments involving simple cell count carried out at 24 and 48 h, showed a dose dependent effect on proliferation (Figure S2A). At 24 h, treatment with 10 mM NaB induced a reduction of 50% over cells not exposed to the HDACi. At 48 h incubation the number of live cells at 10 mM NaB was approximately 10%. H460 cells incubated with NaB at concentrations of 1, 3 and 10 mM were then tested for viability using the MTT assay. The results (Figure S2B) showed a moderate effect at 24 h yielding almost 80% viability at 10 mM NaB and approximately 30% viability after 48 h incubation with the same concentration. Essentially, the same results were obtained when these experiments were repeated with TSA, using concentrations spanning 0.02–1 µM, except that at the highest concentration, TSA seemed to be more toxic than NaB (Figure S2D–E). These results showed that NaB and TSA, although belonging to different chemical classes, shared similar effects. Although the number of cells was clearly reduced as a result of the NaB and TSA action, the main effects of the two inhibitors could be best interpreted as inhibition of proliferation, rather than a direct toxic effect. In order to test whether the treated cells were damaged by the HDACis, lactate dehydrogenase release was assayed after treatment for 24 and 48 h with NaB and TSA (Figure S2F) at several concentrations. Lactate dehydrogenase release was not significantly affected by NaB at 24 h (Figure S2C). At 48 h and only at a concentration of 10 mM did NaB treatment significantly induced lactate dehydrogenase release. Furthermore, inspection of treated cells by fluorescence microscopy after staining with DAPI (Figure S1) revealed intact nuclei and chromatin, a result which would argue against cell damage. Because of the broad spectrum of activities of NaB, experiments were conducted to check whether treatment of H460 cells with this inhibitor could influence cell distribution along the major stages of the cell cycle. These experiments were carried out by quantifying treated and untreated cells by means of fluorescence activated cell sorting. The results in Figure S3A show that following a 24 h treatment, most cells, approximately 80%, were found at the G0/G1 phase of the cell cycle with a concomitant reduction of the S phase. Incubation of cells with 0.2 µM TSA for 24 h produced a similar profile (Figure S3B). Taking into account those results, the treatment of 10 mM NaB for 24 h induced differentiation and inhibition of cell growth, but was not toxic to H460 cells. Thus, experiments involving long term incubation of cells with the HDACis were not extended beyond 24 h.

### Sodium butyrate decreased lactate release of H460 cells, reduced the expression of GLUT 1 and increased GLUT 3 expression

**Figure 2:**
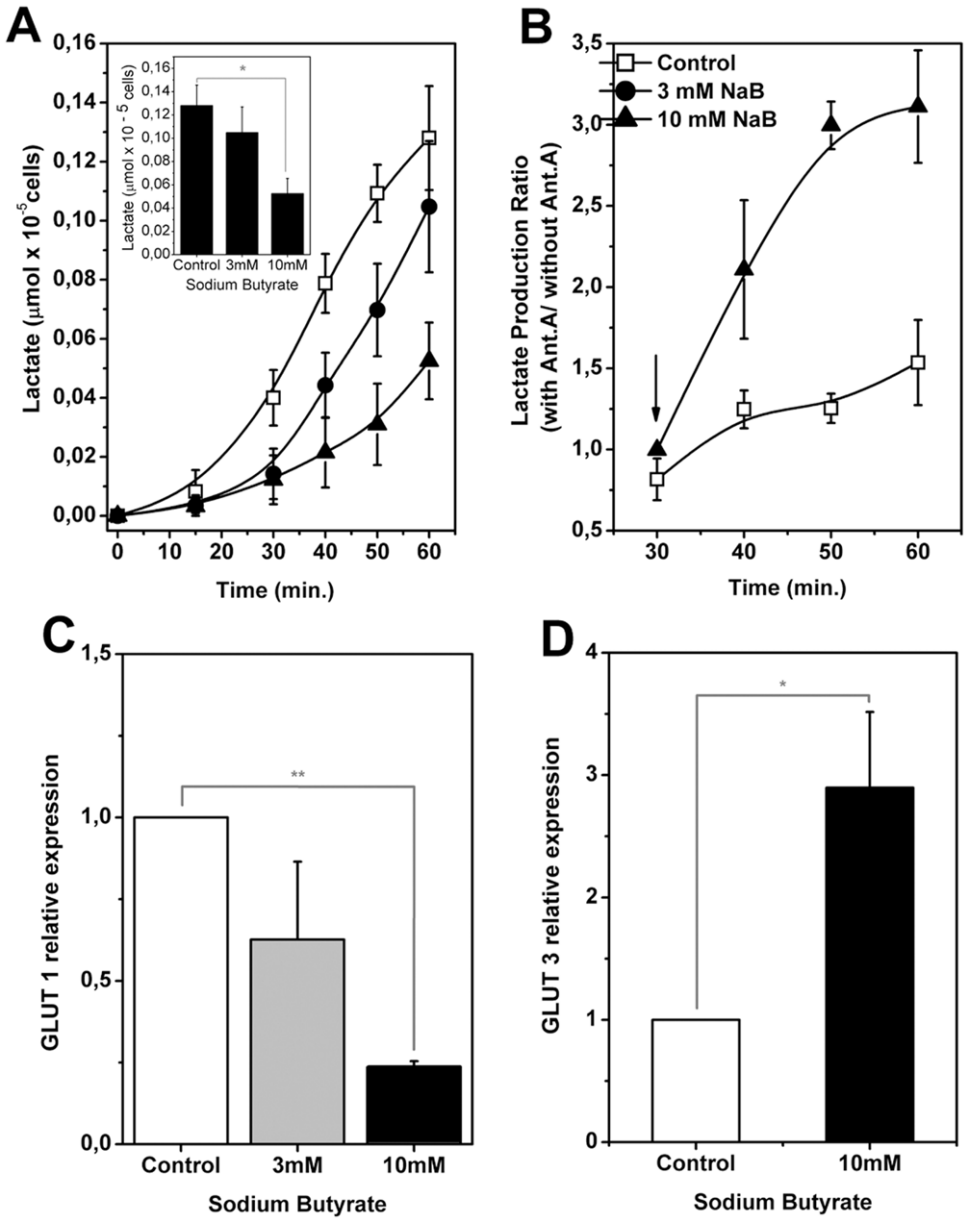
Sodium butyrate reduces lactate production and regulates glucose transporter isoform 1 (GLUT 1) and 3 (GLUT 3) expression. After 24 h of treatment with 3 mM or 10 mM NaB, H460 cells were incubated with glucose-supplemented medium. Aliquots of supernatants were collected every 10 minutes and incubated in hidrazine buffer pH 9.2, with an excess of NAD^+^ and lactate dehydrogenase (LDH) for measurement of lactate released. (A) Kinetics of lactate release and representation of lactate release after 60 minutes (inset). (B) After 30 minutes of incubation with glucose, 2 µg/ml antimycin A was added to the culture. Aliquots of the supernatant were taken at 10 minutes intervals and lactate released was measured. The lactate production ratio of H460 cells in the presence and absence of antimycin A evidences the stimulation on lactate production when oxidative phosphorylation was inhibited by the addition of this drug (indicated by the black arrow). Values represent mean ± SEM; N = 4, *P<0.05. (C) Cells were treated with 3 mM or 10 mM NaB for 24 h and GLUT 1 and (D) GLUT 3 expression was determined by Real Time PCR. Actin was used to normalize cDNA amounts. Values represent mean ± SEM; N = 3, *P<0.05; **P<0.01.

Regarding the energy metabolism, one of the main features of highly proliferative cells, including tumor cells, is their shift to anaerobic glycolysis [3], [20], [21]. The selective pressure, if applicable, producing such an altered phenotype must result from regulatory mechanisms that somehow are able to sense the energy status of the cells. Therefore, as a first step towards uncovering metabolic pathways affected by NaB and TSA, we inquired whether these HDACis could directly affect the glycolytic flux of H460 cells. This series of experiments began by measuring the amount of lactate in a culture medium after cell incubation with 3 and 10 mM NaB for 24 h. The amount of lactate released was then monitored at regular intervals over a period of 60 min. The results are shown in Figure 2A. The values observed in the lactate release were similar to those observed by Pereira da Silva [19]. It can be seen that NaB reduced lactate release in a dose dependent manner. A similar pattern of inhibition of lactate release was obtained after incubation of the cells with 0.2 µM TSA for 24 h (Figure S4A). After 60 min. incubation, TSA-treated cells released approximately 60% of the amount of lactate released by controls. Lactate fluctuations could occur as a consequence of disturbances in any stage of the glycolytic pathway. Taking into consideration that in the present work the experiments were carried out with cells in culture, lactate recycling through gluconeogenesis was ruled out. One possible fate for lactate could be the cells' oxidative metabolism, assuming of course that the mitochondria of the tumor cells were functional. Therefore, lactate release was assayed after incubation of H460 cells with NaB for 24 h followed by addition of antimycin A. The results expressed as the ratio between lactate release in the presence and absence of antimycin A, are shown in Figure 2B. After 60 min. with NaB and antimycin A, lactate release plateaued out exhibiting a twofold increase over the untreated cells. 0.2 µM TSA produced a similar response to antimycin A after 60 min. incubation (Figure S4B). Besides showing that the oxidative metabolism is operational in H460 cells, and supposedly increased in HDACi treated cells, these results also supported the interpretation that NaB and TSA did indeed affect the glycolytic flux. However, glucose uptake by the tumor cells could itself constitute the pacemaker for the entire glycolytic pathway. Therefore, experiments were designed to test whether NaB had any effect on the expression of GLUT 1 and GLUT 3 using qRT-PCR. The results in Figure 2C shows that NaB incubated at 3 and 10 mM during 24 h reduced the expression of GLUT 1 (1.6 and 4, respectively) and increased GLUT 3 expression (2.9X) in H460 cells.

### Sodium butyrate increased mitochondria bound hexokinase activity

**Figure 3:**
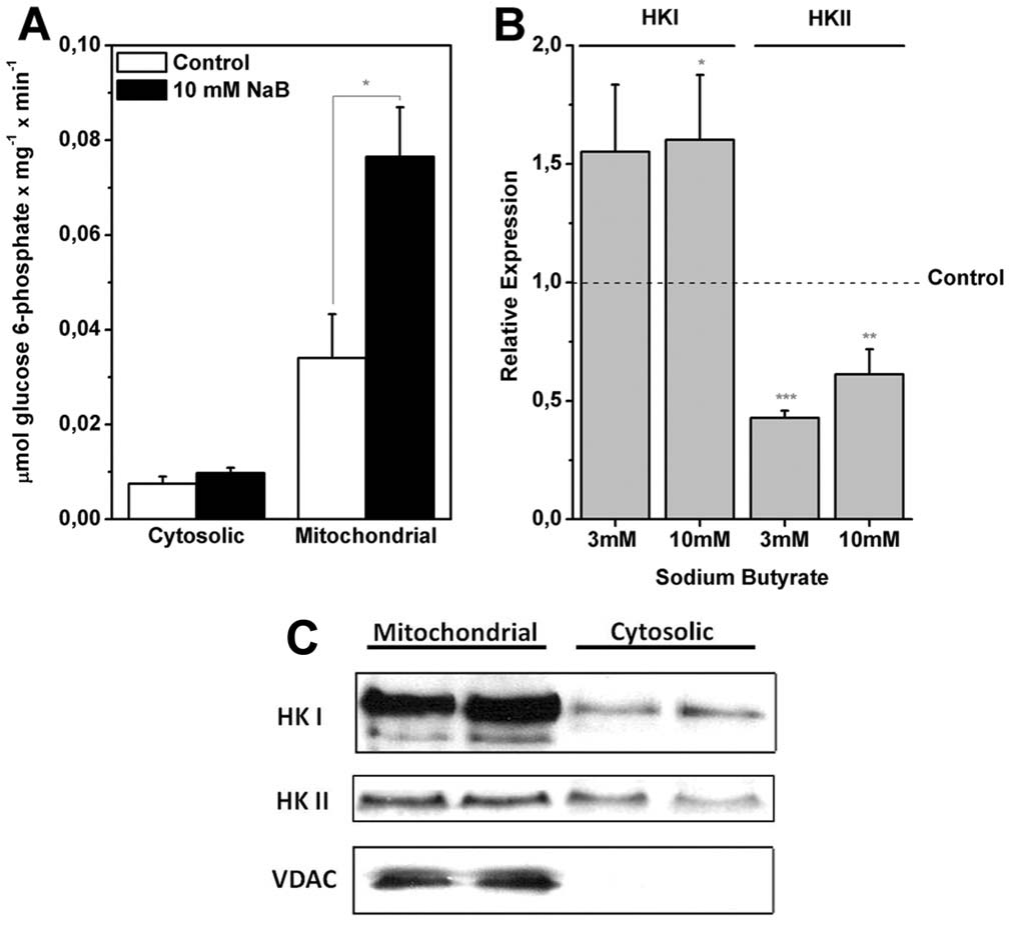
Treatment of H460 cells with sodium butyrate increases mitochondria bound hexokinase (mt-HK) activity and expression. (A) Specific HK activity present in cytosolic and mitochondrial fractions of H460 cells treated or not with 10 mM NaB was assayed using an enzymatic assay coupled to glucose 6-phosphate dehydrogenase. Values represent mean ± SEM; N = 4, *P<0.05. (B) Relative HK I and HK II expression was determined by real time PCR. (C) Western-Blot using anti-HK I, HK II and VDAC primary antibodies. Values represent mean ± SEM; N = 3, *P<0.05; **P<0.01; ***P<0.001.

The observed reduction in GLUT 1 and increased expression of GLUT 3 (Fig 2C) suggested that a compensatory mechanism for glucose uptake was operative in the cells. Thus, we asked whether HK activity, an important element in the kinetics of glucose uptake and glycolytic flux, could play a role. To investigate this, the HK activity was performed and the expression of HK isoforms was evaluated by qRT-PCR. Figure 3A shows that as a result of incubation of H460 cells with 10 mM NaB for 24 h, the activity of mitochondria bound HK increased twice as much as that of the untreated controls. In contrast, NaB did not affect the activity of cytosolic HK. The intracellular location of HK I was also confirmed by immunofluorescence. The results are shown in (Figure S5; Method S2). In these experiments, detection of mitofusin (Mfn) I and II, proteins that are anchored to mitochondria and participate in the maintenance of their morphology and fusion, was carried out in the same preparation so as to provide markers for the organelles. Comparison of the Mfn plates (stained in red) to HK (stained in green), showed that both proteins co-localized in the mitochondria. The higher enzymatic activity observed in Figure 3A could have reflected an increased expression of HK induced after a long term incubation of cells with NaB. This possibility was investigated by assaying HK I and HK II expression using qRT-PCR in H460 cells exposed to 3 and 10 mM NaB for 24 h. The results are shown in Figure 3B. Both concentrations of NaB significantly increased the expression of HK I. Conversely, NaB induced a decrease in the expression of HK II. In the experiments measuring HK II expression (Figure 3B), the values obtained after incubation of cells with 3 and 10 mM NaB were not significantly different. Confirmation that NaB induced an increased expression of HK I was obtained from experiments determining the actual amount of translated HK by means of western blots. The results are shown in Figure 3C. It can be seen that treatment of H460 cells with 10 mM NaB for 24 h clearly increased the intensity and the area of the band corresponding to HK I associated to the mitochondria. However, no alteration was observed in the amount of HKII. In contrast, cytosolic HK I and II were barely detectable in controls and treated cells. The higher amount of HK shown in Figure 3C could not be explained by an increased availability of the mitochondrial hexokinase acceptor, the VDAC, since the amounts of this protein did not change significantly upon treatment with NaB. Whilst the effect of NaB on the expression of HK isoforms was clearly demonstrated, the inhibitor did not affect the activity of either PYK or LDH. The results are shown in Table 1. The fact that 10 mM NaB did not affect these glycolytic enzymes strengthens the idea that the HDACi is partially selective in its effect to modulate the HK activity isoforms. Furthermore, the observation that NaB did not significantly alter the activities of these glycolytic enzymes suggested that the fluctuations leading to lactate production shown in Figure 2A and 2B and Figure S4A and S4B, were a consequence of disturbances at the earlier stages of the glycolytic pathway, rather than at the end.

**Table 1:** Effect of sodium butyrate on recovered piruvate kinase and lactate dehydrogenase activities from H460 cells.

	Specific Activity (µmol x mg^−1^ x min^−1^) (a)	
	**Control**	**NaB**
**Piruvate kinase**	2.91±0.23	2.34±0.27
**Lactate dehydrogenase**	2.59±0.17	2.88±0.14

(a) Specific PYK and LDH activities present in cytosolic fraction of H460 cells was assayed using an enzymatic assay coupled to lactate dehydrogenase. Values represent mean ± SEM; N = 4 for PYK activity and N = 6 for LDH activity.

### Sodium butyrate increased the activity of glucose 6-P-dehydrogenase

**Figure 4:**
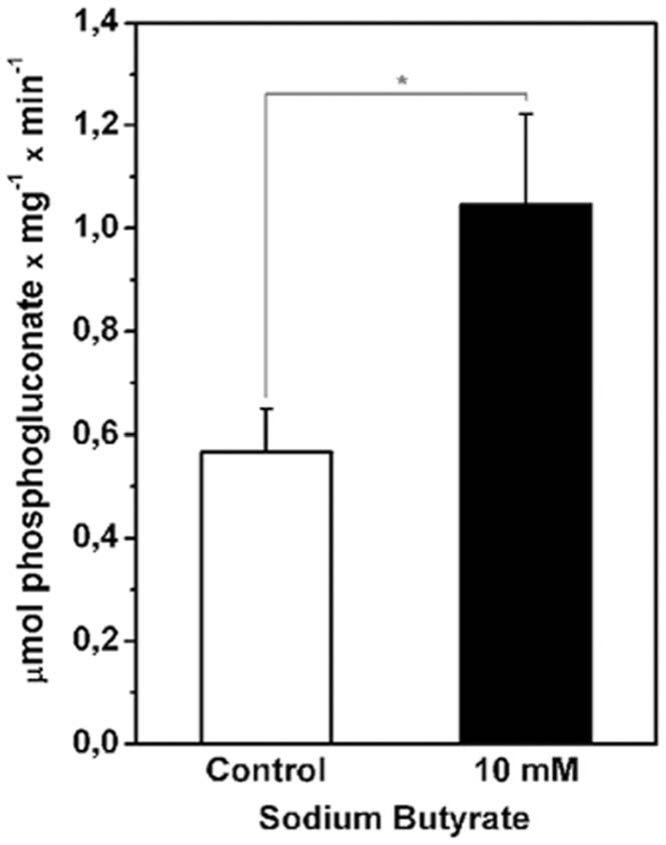
Treatment with sodium butyrate induces an increase in specific glucose-6-phosphate dehydrogenase (G6PDH) activity. H460 cells were treated with 10 mM NaB for 24 h and specific G6PDH activity present in cytosolic fraction was assayed as described under “Experimental Procedures”. Values represent mean ± SEM; N = 5, *P<0.05.

The data showing a NaB induced overexpression of HK and its consequent augmented activity had to contend with the observation that lactate release was diminished as a result of the HDACi action on H460 cells. Because glucose-6-phosphate formed by HK is also a substrate for G6PDH of the PPP. Moreover, Boren and co workers have shown through metabolic control analysis that the glucose-6-phosphate dehydrogenase is one of the main regulators of the PPP [22]. We next inquired whether this sequence of reactions could constitute an alternative sink for the phosphorylated sugar. Thus, G6PDH activity was assayed in H460 cells in the presence and absence of NaB. The results are shown in Figure 4. Incubation with 10 mM NaB did stimulate cytosolic G6PDH activity by almost two fold. This result showed that NaB induced a shunt of glucose-6-phosphate to PPP that can be important to generation of the cell's reductive power.

### Sodium butyrate treatment increased mitochondrial metabolism in H460 cells

**Figure 5:**
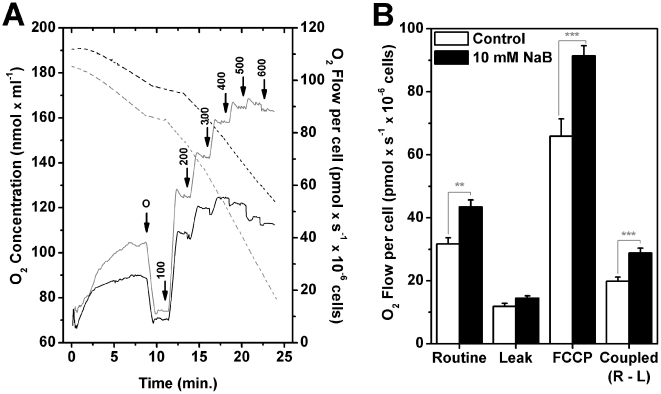
High-resolution respirometry shows an increase in oxidative metabolism subsequent to treatment with sodium butyrate. (A) Representative record of oxygen concentration and flow of intact H460 cells treated or not with 10 mM NaB for 24 h. During the assay, cells were maintained in RPMI medium with glucose and without FBS. Black dashed line represents oxygen concentration in control cells and gray dashed line in NaB treated ones. Black solid line represents oxygen flow in control cells and gray solid line in NaB treated ones. “O” 1 µg/mL oligomycin; “arrows” indicate the titration of FCCP (nM). (B) Effect of NaB treatment on respiratory parameters of intact H460 cells. Routine respiration - basal respiration of H460 intact cells; Leak respiration - rate of oxygen consumption after the addition of oligomycin, that is, respiration not coupled to ATP synthesis; FCCP - maximum respiratory capacity (induced by the addition of FCCP); Coupled respiration - respiration coupled to ATP synthesis, obtained by subtraction of Leak from Routine respiration. Values represent mean ± SEM; N = 9, **P<0.01; ***P<0.001.

The experiments in which antimycin A was used to evaluate lactate release (Figure 2B and S4B) showed that cells treated with NaB or TSA were more susceptible to oxidative phosphorylation inhibition and, therefore, responded by increasing the glycolytic flux and the amount of lactate released when compared to controls. In view of the potential inclusion of mitochondria as targets for NaB, the next experiments described the attempts to directly measure respiratory parameters in the presence of the HDACi. High-resolution respirometry allowed the detection of small changes in O_2_ flux in the electron transport system coupled or not to proton gradient and required lower numbers of cells. Initially, O_2_ consumption rates were measured in intact H460 cells treated or not with NaB for 24 h. The results are shown in Figure 5A. A significant increase in routine respiration (in RPMI medium containing 11.1 mM glucose) was observed with approximately 42% enhancement induced by 10 mM NaB after 24 h incubation. The addition of oligomycin allowed the evaluation of O_2_ consumption rate uncoupled to ATP synthesis referred to as leak respiration. Although NaB did not promote any effects on leak respiration, O_2_ consumption related to ATP synthesis (i.e., coupled respiration = routine respiration – leak respiration), was increased by 45% in treated cells (Figure 5B). NaB treatment also promoted a significant increase in O_2_ consumption when mitochondria were at maximum respiratory capacity, as indicated by the results of experiments in which the proton ionophore carbonyl cyanide 4-(trifluoromethoxy) phenylhydrazone (FCCP) was added, as shown in Figure 5B. A similar pattern of increase in oxidative metabolism was obtained after incubation of the H460 cells with 0.2 µM TSA for 24 h (Figure S6). The effects of NaB on O_2_ consumption on H460 cells could result from NaB catabolism itself. In order to address this point, 10 mM NaB was directly added to the oxygraph chamber and O_2_ consumption was measured in H460 cells grown without prior addition of NaB for 30 min. No significant increments in O2 consumption were observed, under those conditions (data not shown). As a result of the enhanced O_2_ consumption, parallel experiments were conducted in order to show whether this pattern was accompanied by an increase in ATP content of the treated cells. The result in Table S2 (Method S1) showed that there was no significant alteration in cellular ATP content upon treatment with the HDACi. Although such a result would indicate that the mitochondria are fully functional, the possibility that the ATP detected originated in the glycolytic pathway or for that matter from any other ATP producing reaction cannot be excluded.

### Sodium butyrate abolished the Crabtree effect

**Figure 6:**
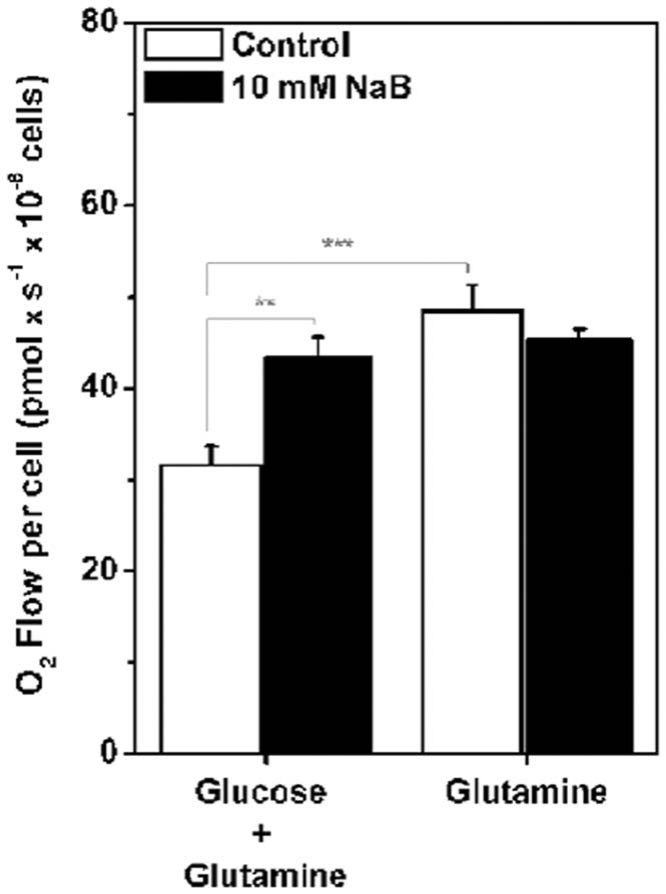
The crabtree effect is not observed in H460 cells after sodium butyrate treatment. After treatment with 10 mM NaB for 24 h, H460 cells oxygen flow was measured on DMEM medium with or without glucose containing 2 mM glutamine as respiratory substrate. This result confirms the stronger oxidative metabolic profile of H460 cells treated with NaB. Values represent mean ± SEM; N = 5, **P<0.01; ***P<0.001.

Glutamine not only generates glutathione for the maintenance of the cell's reductive power, but also feeds anaplerotic pathways providing intermediates of the TCA cycle such as glutamate and α-ketoglutarate. In parallel, these reactions also produce NADPH. Hence, experiments using high-resolution respirometry, were carried out to test whether NaB could affect O_2_ consumption by intact H460 cells in the presence of 11.1 mM glucose, or glutamine alone. The results are shown in Figure 6. Untreated H460 cells exhibited a significant increase in routine respiration when glucose was removed from the medium, a response, known as the Crabtree effect (Figure 6, open bars). Interestingly, in NaB treated cells, this effect was not observed (Figure 6, black bars). This result strengthens the hypothesis that the HDACis target the glycolytic pathway and it might reflect yet another feature of the differentiation process promoted by NaB. In parallel these results also reinforced the notion that the mitochondria of H460 cells appear to be fully functional and that NaB-treated cells displayed a more oxidative phenotype.

### Sodium butyrate treatment increased mitochondrial respiration in complex II of permealized H460 cells and succinate dehydrogenase activity

**Figure 7:**
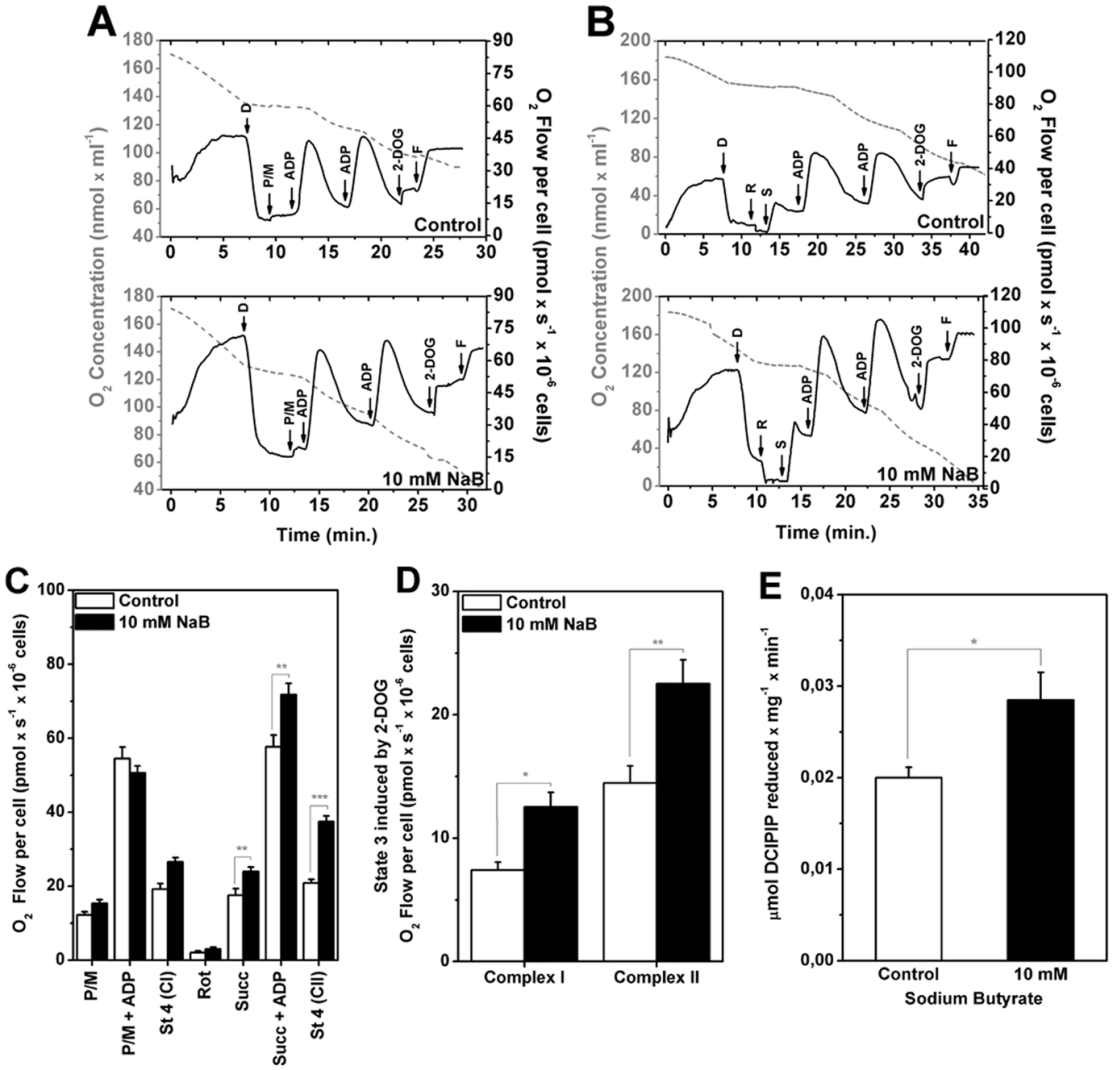
Sodium butyrate treatment increases oxygen consumption when succinate is used as respiratory substrate. H460 cells were treated with NaB for 24 h, permeabilized with 0.003% digitonin “D” and oxygen flow or concentration were measured using high-resolution respirometry on respiration buffer. The substrates and/or modulators were added in the following order: 10 mM pyruvate + malate “P/M”, 100 µM ADP, 100 µM ADP, 10 mM 2-deoxy-D-glucose “2-DOG” and 200 µM FCCP “F” or 1 µM rotenone “R”, 10 mM succinate “S”, 100 µM ADP, 100 µM ADP, 10 mM 2-deoxy-D-glucose “2-DOG” and 200 µM FCCP “F”. (A) and (B) Representative assays of oxygen concentration (gray dashed line) and oxygen flow (black solid line) of permeabilized H460 cells. (C) Rate of O_2_ flow per cell after addition of the different substrates and/or modulators. Values represent mean ± SEM (N = 7 – Control; N = 10–10 mM NaB). **P<0.01; ***P<0.0001. (D) Hexokinase bound mitochondria activity stimulates oxygen consumption in NaB treated H460 cells. The stimulus of oxygen consumption was measured as the difference between O_2_ flow per cell in presence of glucose analogue 2-deoxy-D-glucose (2-DOG) and in the absence of ATP synthesis (state 4), in a high-resolution oxygraph. Values were expressed as mean ± SEM. N = 7, * P<0.05; **P<0.01. (E) Specific SDH activity present in mitochondrial fraction of H460 cells treated or not with 10 mM NaB was assayed using an enzymatic assay based on the PMS-mediated reduction of DCPIP. Values were expressed as mean ± SEM. N = 6, * P<0.05.

The effect of NaB to promote an increase in O_2_ consumption in intact H460 cells could be better evaluated by directly analyzing the redox activity of their electron transport system. For example, complex I or II activities could be analyzed in digitonin-permeabilized H460 cells treated or not with NaB using high resolution respirometry. In this assay, one group of cells received pyruvate/malate as substrates for complex I. Another group of cells was treated with rotenone (complex I inhibitor) followed by the addition of succinate as a substrate for succinate dehydrogenase (SDH) respiratory complex II. The results in Figure 7 show that whereas there was no NaB associated effect on cells that received pyruvate/malate, the HDACi increased O_2_ consumption when succinate was used as respiratory substrate, suggesting a higher activity of mitochondrial transport system associated to SDH. The analysis of SDH activity confirmed this increase in mitochondrial complex II (Figure 7E). It has been shown elsewhere that SDH activity prevented the pseudo-hypoxia that leads to HIF stabilization and its downstream adaptative responses [23], [24] commonly observed in several types of cancer. Such result indicated that SDH may have acted as a tumor suppressor (Figure 7B and C).

Collectively the results shown here (Figures 3A, 5A and B) suggested that the increase in mitochondrial respiration and mt-HK I activity could be coupled as was demonstrated in brain tissues which normally displays a high energy demand [25]. However, no mechanistic link between those two systems has been established in lung cancer cells. The experiments that were designed to fill this gap involved the incubation of cells with 10 mM NaB for 24 h followed by the direct measurements of O_2_ consumption in permeabilized H460 cells in the presence of the glucose analogue 2-deoxyglucose (2-DOG). 2-DOG was used because its phosphorylated form 2-DOG-6P it is not a strong inhibitor of mt-HK catalyzed reaction as occurs with Glc-6P. Thus 2-DOG mimics a steady state physiological glucose phosphorylation in cells [25], [26]. The results are shown in Figure 7D. In both experiments with complex I or II linked substrates, NaB promoted higher O_2_ consumption than in the untreated control cells. A plausible explanation for these findings invokes the previous results showing that NaB stimulates HK I expression and HK activities (Figure 3A–C). Because HK I is able to phosphorylate 2-DOG, this in turn could supply the ADP necessary to boost the oxidative phosphorylation occurring within the mitochondria as shown in Figure 7D.

### NaB did not induce mitochondria biogenesis

**Figure 8:**
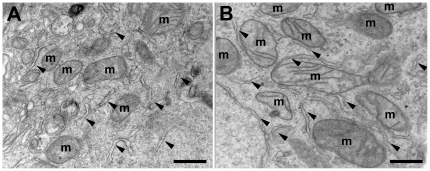
Sodium butyrate treatment alters mitochondria structure in H460 cells. Electron microscopy of longitudinal sections of (A) H460 cells control and (B) treated with 10 mM NaB for 24 h. Bars: 1 µm. Arrowheads show the proximity between endoplasmic reticulum and mitochondria.

Considering that an increase in cell oxidative metabolism usually correlates with an increase in the number of mitochondria in the cell we decided to investigate if NaB treatment induced mitochondrial biogenesis. Mitochondrial biogenesis was investigated using two different approaches: quantifying mitochondrial DNA in relation to nuclear DNA as well as by assaying CS activity. The results presented in Table 2 show that the copy number of mitochondria DNA of control cells did not differ significantly from that of NaB treated cells. A similar result was obtained with CS activity. These results suggested that the treatment with 10 mM NaB during 24 h did not induce mitochondria biogenesis. In spite of these results, NaB was shown to have an effect on the expression of Mfn, also shown in Table 2. There was a significant increase in the expression of this protein, indicating that rather than affecting mitochondrial biogenesis, the higher amount of Mfn detected here may be involved with tethering between functionally distinct organelles, such as the endoplasmic reticulum [27] as well as mitochondria themselves. Attempts to verify this possibility involved the examination by electron microscopy of mitochondria obtained from cells treated with 10 mM NaB. The results are shown in Figure 8. Analysis of the plates did not allow any conclusion regarding the occurrence of a higher frequency of bridging between mitochondria (m) and ER (Figure 8, arrowhead), or for that matter any other recognizable cellular structure. However, the most obvious alteration induced by NaB was the presence of mitochondria that were more elongated with a higher resolution of the cristae (Figure 8) in comparison with controls. The increased expression of Mfn and the appearance of more elongated mitochondria in the H460 cells after treatment, suggest that NaB could induce a mitochondrial fusion.

**Table 2:** Effect of sodium butyrate on mt-DNA content, citrate synthase activity and mitofusin I relative expression.

	Control	NaB
**mt-DNA** (a)	1.00	1.28±0.13
**Citrate synthase** (b)	0.68±0.15	0.80±0.15
**Mitofusin I** (c)	1.00	1.40±0.1*

(a) Mitochondrial DNA quantification (mt-DNA/nDNA). Total DNA (cytosolic and mitochondrial) was extracted from control and NaB treated cells and NADH-dehydrogenase subunit I (a gene coded by the mitochondrial genome) content was estimated by real-time PCR analysis. Actin DNA (a nuclear coded gene) was used to normalize DNA amounts. Values represent mean ± SEM; N = 4.

(b) Specific citrate synthase activity (µmol x mg^−1^ x min^−1^) present in mitochondrial fraction of H460 cells treated with 10 mM NaB for 24 h. Values represent mean ± SEM; N = 5.

(c) Mitofusin I mRNA relative expression. Actin was used to normalize cDNA amounts. Values represent mean ± SEM; N = 3, *P<0.05.

### Sodium butyrate altered the glycolytic metabolite profile of intact H460 cells

**Figure 9:**
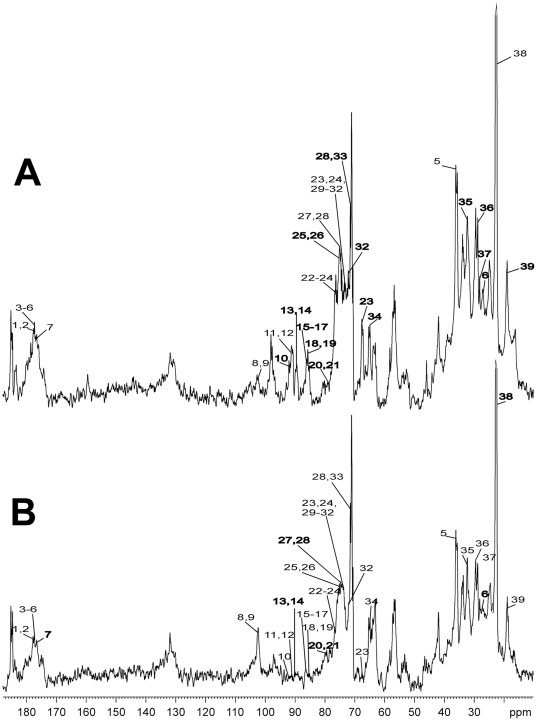
Metabolomics profile by NMR spectroscopy. (A) Control H460 cells or (B) cells treated for 24 h with 10 mM NaB were incubated in DMEM containing ^13^C-glucose. After the incubation, cells were harvested, and intact cells were analyzed by NMR spectroscopy. Spectra highlight the enrichment of the following ^13^C-containing informative metabolic intermediates: (1) Oxoglutaric acid; (2) Citric acid; (3) Fumaric acid; (4) L- Asparagine; (5) L-Glutamic acid; (6) L-Lysine; (7) L-Leucine; (8) 5-Methylcytidine; (9) 5-Methyldeoxycytidine; (10) Uridine; (11) Deoxyinosine; (12) Deoxyguanosine; (13) Coenzyme A; (14) 2-Acetolactate; (15) dGDP; (16) dGTP; (17) NAD; (18) NADPH; (19) NADP; (20) Phosphoribosyl pyrophosphate (PRPP); (21) Sedoheptulose 7-phosphate; (22) Xylulose 5-phosphate; (23) Fructose 6-phosphate; (24) Gluconolactone; (25) Cytidine triphosphate; (26) Cytidine monophosphate; (27) 2-Phospho-glyceric acid; (28) Glucose 6-phosphate; (29) 6-Phosphonoglucono-lactone; (30) Glyceric acid 1,3-biphosphate, (31) Malic acid; (32) 6-Phosphogluconic acid; (33) Erythrose 4-phosphate; (34) Ribulose 5-phosphate; (35) Acetoacetic acid; (36) L-Glutamine; (37) L-Cysteine; (38) L-lactate; (39) L-Alanine.

In order to evaluate whether the changes in mitochondrial respiration and glucose oxidation were somehow involved in other adaptive pathways of energy metabolism, ^13^C–NMR analysis of the cells treated or not with NaB was carried out with intact cells. The analysis of spectra shown in Figure 9 and Table 3 revealed that NaB treatment promoted several changes on H460 cells metabolic intermediates, a pattern suggestive of a significant metabolic reprogramming. The largest differences were observed in spectral region from 70 to 105 p.p.m. The contents of coenzyme A (13) and 2-acetolactate (14) (involved in coenzyme biosynthesis and pantothenate metabolism) were practically absent in NaB treated-cells, indicating an under structuring process leading to increased oxidative metabolism and confirming the respirometric analysis experiments. Additionally, NaB treatment promoted a severe decrease in the content of metabolites involved in pirymidine metabolism, including uridine (10), deoxyinosine (11), deoxyguanosine (12), dGDP (15), dGTP (16), cytidine triphosphate (25) and cytidine monophosphate (26), in agreement with cell cycle arrest shown in Figure S3A. On the other hand, the contents of 5-methyl deoxycitidine (9) and 5-methylcytidine (8) (spectral region 102 to 103 p.p.m.) were greatly elevated in cells treated with NaB, a result compatible with an anabolic status. Surprisingly, the content of NAD, NADP and NADPH decreased in NaB-treated cells. Since the synthesis of several metabolites depends on nicotinamide metabolism, it is plausible that NaB treatment could influence a wide range of anabolic or catabolic pathways.

**Table 3:** Effect of NaB on the metabolites content in H460 cells.

	METABOLITES CONTENT	
	INCREASE	DECREASE
**Aminoacid and oxidative metabolism**	-----	Coenzyme A (13), 2-acetolactate (14); NAD (17), NADPH (18), NADP (19); leucine (7), asparagine (4), glutamate (5), lysine (6), cysteine (37), alanine (39); oxoglutaric acid (1), citric acid (2), fumaric acid (3), malic acid (31), acetoacetic acid (35);
**Pyrimidine and purine metabolism**	5-methylcytidine (8), 5-methyl deoxycitidine (9); PRPP (20);	Uridine (10), deoxyinosine (11), deoxyguanosine (12), dGDP (15), dGTP (16), cytidine triphosphate (25), cytidine monophosphate (26);
**Glycolysis**	-----	glucose-6-phosphate (28), fructose-6-phosphate (23), 1,3-biphosphoglycerate (30), 2-phosphoglycerate (27), lactate (38);
**Pentose Phosphate Pathway**	sedoheptulose-7-phosphate (21), PRPP (20);	6-phosphogluconolactone (29), 6-phosphogluconate (32), ribulose-5-phosphate (34), xylulose-5-phosphate (22), erytrose-4-phosphate (33); NADP (19);

Inspection of the ^13^C chemical shift from 70 to 80 p.p.m., showed that NaB treatment promoted a decrease in glycolytic intermediates glucose-6-phosphate, fructose-6-phosphate, 1,3-biphosphoglycerate (1,3BPG), 2-phosphoglycerate (2-PG). In agreement with the previous (Figure 2A and B) results, the intracellular lactate content in NaB treated cells, was also decreased as shown by the NMR spectra. The decrease in GLUT1 and increase in GLUT3 transcripts in H460 treated-cells shown in Figure 2C indicated that glucose uptake and utilization reflect the energy requirements of the cells subjected to sodium butyrate treatment. The level of a metabolic intermediate is a result of the balance between its rate of synthesis and consumption by the downstream step. Thus, a decrease in a metabolite, for example, can be result of an inhibition in its upstream formation step or an increase in its downstream degradation step. Therefore, this decrease in several glycolytic intermediates suggests a decreased flux through the glycolytic pathway because lactate release and production is low (Figures 2 and 9). The reduction in glucose-6-phosphate and fructose-6-phosphate contents could be explained by the significant increase in glucose-6-phosphate dehydrogenase activity (Figure 4), which would channel glucose carbons to the PPP. Since the committed step in PPP is the reaction catalyzed by G6PDH, it can be assumed that the flux through the oxidative branch of this pathway is increased. This idea was corroborated by the decrease in NADP content, a metabolite which functions as a co-substrate of G6PDH. Figure 9 and Table 3 also show alterations in PPP intermediates due to NaB treatment. Contents of 6-phosphogluconolactone, 6-phosphogluconate, ribulose-5-phosphate, xylulose-5-phosphate and erytrose-4-phosphate were decreased and of sedoheptulose-7-phosphate increased. Additionally, an increase in phosphoribosyl pyrophosphate (PRPP) content upon treatment was observed. The decrease in 6-phosphogluconolactone, 6-phosphogluconate, ribulose-5-phosphate and xylulose-5-phosphate agrees with the idea of an increased carbon flux through PPP oxidative branch. The increase in sedoheptulose-7-phosphate might indicate alterations in the equilibrium of transaldolase and transketolase reactions, favoring sedoheptulose-7-phosphate accumulation. This is also compatible with the decrease in fructose-6-phosphate and erythrose-4-phosphate. Lastly, the increase in PRPP content could be explained by the suppression of purine and pyrimidine metabolism in NaB-treated cells, this result is in agreement with the reduction in S phase of cell cycle (Figure S3A).

A shift towards an oxidative phenotype induced by NaB treatment was also supported by the decrease in the contents of some L-aminoacids (leucine, asparagine, glutamate, lysine, cysteine, alanine; ^13^C chemical shift 179 to 176 p.p.m. and 63 to 53 p.p.m. and 29 to 19) and some tricarboxilic acid intermediates (oxoglutaric acid, citric acid, fumaric acid and malic acid) and related intermediate (acetoacetic acid).

## Discussion

Whether sporadic or hereditary, most if not all types of cancer ultimately derive from single cells that have undergone irreversible biochemical reprogramming. The phenotypes acquired by the clones of transformed cells are such that the intrinsic pathways normally acting as safeguards for the tissue and the organism become subverted and/or abrogated. The phenotype that confers virtually limitless replication to the transformed cells is costly in terms of energy. In rapidly growing tumors, the prevalent anabolism must be accompanied by upregulated pathways that ultimately increase the rate of ATP synthesis for all processes connected to growth and invasiveness and hence necessarily involve elements of the intermediary metabolism. To make matters even more complex, it is known that the metabolic reprogramming exhibited by transformed cells is not homogeneously distributed throughout the tumor. Cells located at the centre of the tumor mass are under more severe anoxic conditions than those at the periphery and consequently two or more populations are formed that can be loosely classified as aerobic and anaerobic tumor cells depending on their location in this O_2_ gradient. Within a tumor, the mixed cell population of hypoxic and normoxic cells exchange metabolites between each other establishing a network of complementary pathways that collectively have been termed biochemical symbiosis [28]. In this situation it can be inferred that mitochondria of at least part of the cell population are functional. In the present paper we confirmed that aerobic glycolysis and oxidative metabolism coexist in tumor cells and most likely complement each other through complex interactions and that NaB and TSA seem to disturb this energetic equilibrium. We show for the first time that these HDACis reduce the glycolytic metabolism and increase O_2_ consumption coupled to ATP synthesis in H460 cells. In this scenario, the HDACis action transcend their role at the chromatin level because non-histone proteins can be acetylated and most intermediate metabolic enzymes are acetylated, including enzymes of glycolysis, fatty acid metabolism and Krebs cycle [29].

Initially, whatever metabolic reprogramming occurred upon treatment of the cells with NaB, no gross morphological changes were observed at the level of light and electron microscopy. Likewise, the nuclear structure of treated cells was preserved, which makes it improbable that NaB had any disruptive effects on cell architecture, including intracellular compartmentation. In agreement with this view, it is worth mentioning that any known direct interaction of NaB with the cells seems to be receptor mediated, involving, for example solute transporters such as monocarboxylate transporter SMCT1 (SLC5A8) [30], [31]. Incidentally, it has been reported that SMCT1 is usually silenced in cancer cells, a fact that may explain why relatively high concentrations of butyrate had to be used in the present work and in the literature [32]. Indeed, TSA which is readily absorbed by the cells [33] exerted its inhibitory effects at much lower concentrations than NaB. Other issues relating to solute transport through the membranes of H460 cells may have a direct bearing on the results involving lactate efflux. We showed that H460 cells treated with NaB and TSA displayed a diminished lactate release (Figure 2A and S4A), a result which could have reflected a direct inhibitory effect of the HDACi on any of the enzymes participating directly or indirectly in glycolysis. This excluded lactate dehydrogenase, which was shown here to be unaffected by the NaB. In order to maintain a high rate of glycolysis, it is mandatory for the tumor cells to have access to a ready supply of glucose. In many types of cancers, glucose transport is performed by class 1, 3 and 4, which as a rule can be overexpressed in tumor cells. It has been suggested that GLUT 1 and GLUT 3 are regulated by activation of HIF-1α [5], [34]. In the present work we showed that NaB treatment, particularly at 10 mM, strongly inhibited the expression of GLUT 1 and increased GLUT 3 expression (Figure 2C) in H460 cells, a result which suggest that a compensatory mechanism for glucose uptake is taking place. GLUT 1 is present in a variety of tissues that sense and respond to fluctuations in blood glucose levels. Our results (Figures 2 and 3) indicated that HDACi effects on GLUT and HK in H460 is similar to that of brain cells. In this context, Gould and Holfman suggested that under normal conditions the capacity of HK to phosphorylates glucose (the main energy source) is considerably greater than the capacity of the glucose transport systems in brain cells. However, under conditions of either high glucose demand or hypoglycemia, the expression of GLUT 3 in the brain with a low Km for hexoses may be required as an ancillary transport system [35].

Upon entering the cell after the GLUT 1 barrier, glucose is immediately phosphorylated and thus initiates the glycolytic pathway. In H460 cells, HK associated to the mitochondria was found to be overexpressed as a consequence of NaB treatment (Figure 3A). The question remained as to which HK isoform responded to the HDACi. This question was addressed by real time PCR which revealed that isoform HK I was upregulated and HK II down regulated by NaB (Figure 3B). Upregulation of HK I was rather surprising and raised some points for speculation. For example, how did this finding fit with the general NaB induced depression of glycolysis reflected by the diminished lactate efflux? This question could be answered, at least partially, by highlighting the results in Figure 4 that show clearly that NaB was able to stimulate the activity of G6PDH indicating that G6P produced by HK I could be diverted to the PPP. The fate of G6P as a substrate to G6PDH also explains why G6P did not feedback inhibit HK I activity. In addition, activation of the PPP would provide a salvage pathway for anabolic metabolites in parallel with NADPH formation as a co adjuvant for reductive synthesis.

Admittedly, other enzymes of the glycolytic pathway that were not investigated in the present work might have played key roles in the overall effects produced by the HDACis. One such example is hexose phosphate isomerase (HPI) which has been recently shown to play an important modulatory activity in glycolysis using kinetic models [36]. Because HPI can directly affect both, G6P and fructose-6-phosphate (F6P) concentrations, and simultaneously be subject to inhibition by fructose 1,6-bisphosphate and PPP intermediaries, its response to HDACis could perhaps explain some of the observations described here. These experiments are currently under way.

If glutamine catabolism is representative of the status of mitochondria of H460 cells, one could conclude that as a whole the organelle seems to be fully functional. As a matter of fact, we observed that NaB stimulated mitochondrial metabolism of H460 cells by measuring several parameters which collectively could be summarized as an enhancement of O_2_ consumption associated to ATP synthesis (Figure 5). For instance, a large increase in respiration of NaB-treated, permeabilized cells to which 2-DOG was added (Figure 7D) support the idea that the increased routine respiration in intact cells (Figure 5) is due to an increase in the HK activity bound to mitochondria. In this pro-oxidant scenario, the ADP produced by high HK activity could control both membrane potential and ROS generation through an ADP-recycling mechanism similar to that proposed by Da-Silva et al [25].

In connection with the mechanism of NaB activation of respiration, various interpretations, not necessarily mutually excluding, are possible. In one scenario, there could be a direct interaction of the HDACi with any of the mitochondrial components, similarly to what was described for HK. Conversely, NaB could act elsewhere and ultimately affect the respiratory parameters of the mitochondria. Perhaps the broadest consideration that must be taken into account is the possibility that most if not all the HDACis effects described in the present work could have been brought about by an action on a central factor that, in turn, would affect other pathways. One possibility is that HDACis are decreasing HIF-1α, and this effect decreases glycolysis maintained by HIF-1α. A key transcription factor that knowingly acts as the hub for many processes associated to the cell cycle and the energy metabolism itself. Thus, by preferentially targeting HIF-1α, NaB or TSA could indirectly affect hundreds of other genes. At any rate, investigation of the effect of HDACis on HIF-1α regulation and on key actors of anaerobic and oxidative metabolism is currently under way.

Although more evidence has yet to be procured, the set of results contained herein suggests that NaB and TSA could induce H460 cells to assume an altogether differentiated state. It is proposed that in this differentiated phenotype, cells actually shift towards more oxidative metabolism akin to untransformed cells. Ultimately, the enhanced oxidative metabolism would harm the H460 cells by way of excessive production of ROS that can cause cell death. In this context, the H460 cells treated with NaB could activate antioxidant defense mechanisms related to the increase in: (a) HK activity bound to mitochondria (Figure 3A), which increases ADP recycling; (b) G6PDH activity (Figure 4), which increases PPP flux and NADPH supply; and (c) the expression of Mfn 1 (Table 2), which, may reflect the initial stages of a process of mitochondrial fusion that can ultimately act as inhibitor of apoptosis. Indeed it has been shown that under oxidative stress mitochondria can rescue their membrane potential [37]. Although the notion that NaB and TSA induced generation of ROS is a reasonable proposition, it must be mentioned that the topic of ROS and cancer cells is in itself quite controversial. There are data supporting the view that ROS are in fact essential requirements for the survival of tumor cells [38], [39], even though the mechanism whereby ROS would support tumor growth remains elusive. Taken together the results presented here revealed a unique biochemical profile induced by NaB and TSA. The data essentially showed that alterations in the glycolytic flux triggered an enhancement of mitochondrial function that was not, however, paralleled by cell proliferation. Whilst further studies are paramount to unveil the mechanisms underlying the interplay between glycolysis and oxidative metabolism, the groundwork established in the present work already suggests that the stimulation of the oxidative metabolism in tumor cells may be an interesting strategy for chemotherapy.

## Supporting Information

Figure S1 **Sodium butyrate induces F-actin rearrangements in H460 cells.** H460 cells were gown in the absence (A) or presence (B) of 10 mM NaB. Cells were labeled with rhodamine-conjugated phaloidin (red) and DAPI (blue), to allow visualization of actin citoeskeleton and nuclei, respectively. Representative immunofluorescence images obtained using a Zeiss AxioObserver Z1 are shown. Bars: 30 µm.
(TIF)

Figure S2 **Sodium butyrate and trichostatin A induce time and dose-dependent inhibition of growth in H460 cells.** Cells were grown in the absence or presence of various concentrations of NaB (1, 3 and 10 mM; A–C) or TSA (0.02, 0.2 and 1 µM; D–F) for 24 or 48 h. At indicated times, cell number and viability were analyzed by **t**rypan blue exclusion (A and D) and MTT assay (B and E)**.** Also, citotoxicity of the different amounts of NaB or TSA was estimated by measurement of lactate dehydrogenase (LDH) release using CytoTox96 Non-Radioactive Cytotoxicity Assay Kit (Promega) (C and F). Values represent mean ± SEM; N = 3, *P<0.05; **P<0.01; ***P<0.001.
(TIF)

Figure S3 **Sodium butyrate and trichostatin A induces cell cycle arrest and inhibits cellular DNA synthesis.** H460 cells were treated or not with 3 or 10 mM NaB (A) and 0.2 µM TSA (B) for 24 h. Posteriorly cells were harvested, permeabilized, incubated with propidium iodide and had its DNA content analyzed by flow cytometry using FACScan. Values represent mean ± SEM; N = 4, *P<0.05; **P<0.01; ***P<0.001.
(TIF)

Figure S4 **Trichostatin A reduces lactate production in H460 cells.** After 24 h of treatment with 0.2 µM TSA, H460 cells were incubated with glucose-supplemented medium. Aliquots of supernatants were collected every 10 min. and incubated in hidrazine buffer pH 9.2, with an excess of NAD^+^ and lactate dehydrogenase (LDH) for measurement of lactate release. (A) Kinetics of lactate release and representation of lactate release after 60 minutes (inset). (B) After 30 minutes of incubation with glucose, 2 µg/ml antimycin A was added to the culture. Aliquots of the supernatant were taken at 10 minutes intervals and lactate released was measured. The lactate production ratio of H460 cells in the presence and absence of antimycin A evidences the stimulation on lactate production when oxidative phosphorylation was inhibited by the addition of this drug (indicated by the black arrow). Values represent mean ± SEM; N = 4, *P<0.05.
(TIF)

Figure S5 **HK I is bound to mitochondria in H460 cells.** Control (A and C) or NaB treated (B and D) H460 cells were labeled with antibodies against mitofusin I (A–B) or mitofusin II (C–D) (red), Hexokinase I (green). Merge HK + Mfn (yellow) and DAPI (Blue). Representative images taken with an inverted fluorescence microscope Zeiss AxioObserver Z1 are shown. Bars: 30 µm.
(TIF)

Figure S6 **High-resolution respirometry shows an increase in oxidative metabolism subsequent to treatment with trichostatin A.** (A) Representative record of oxygen concentration and flow of intact H460 cells treated or not with 0.2 µM TSA for 24 h. During the assay, cells were maintained in RPMI medium with glucose and without FBS. Black dashed line represents oxygen concentration in control cells and gray dashed line in TSA treated ones. Black solid line represents oxygen flow in control cells and gray solid line in TSA treated ones. “O” 1 µg/mL oligomycin; “arrows” indicate the titration of FCCP (nM). (B) Effect of TSA treatment on respiratory parameters of intact H460 cells. Routine respiration - basal respiration of H460 intact cells; Leak respiration - rate of oxygen consumption after the addition of oligomycin, that is, respiration not coupled to ATP synthesis; FCCP - maximum respiratory capacity (induced by the addition of FCCP); Coupled respiration - respiration coupled to ATP synthesis, obtained by subtraction of Leak from Routine respiration. During the assay, cells were maintained in RPMI medium with glucose and without FBS. Values represent mean ± SEM; N = 5, *P<0.05, **P<0.01; ***P<0.001.
(TIF)

Table S1 **List of** **primer sequences used for quantitative Real Time PCR.**
(DOC)

Table S2 **Effect of sodium butyrate on ATP content in H460 cells.** ATP content was assayed using an enzymatic method with hexokinase and glucose-6-phosphate dehydrogenase [40]. Values represent mean ± SEM; N = 3.
(DOC)

Method S1 **ATP Content.**
(DOC)

Method S2 **Immunocytochemistry.**
(DOC)
