# Supplementary figures and images for: Energy Metabolism in H460 Lung Cancer Cells: Effects of Histone Deacetylase Inhibitors
Source: PLoS One. 2011 Jul 18;6(7):e22264. doi: 10.1371/journal.pone.0022264 (PMC3138778; doi:10.1371/journal.pone.0022264)

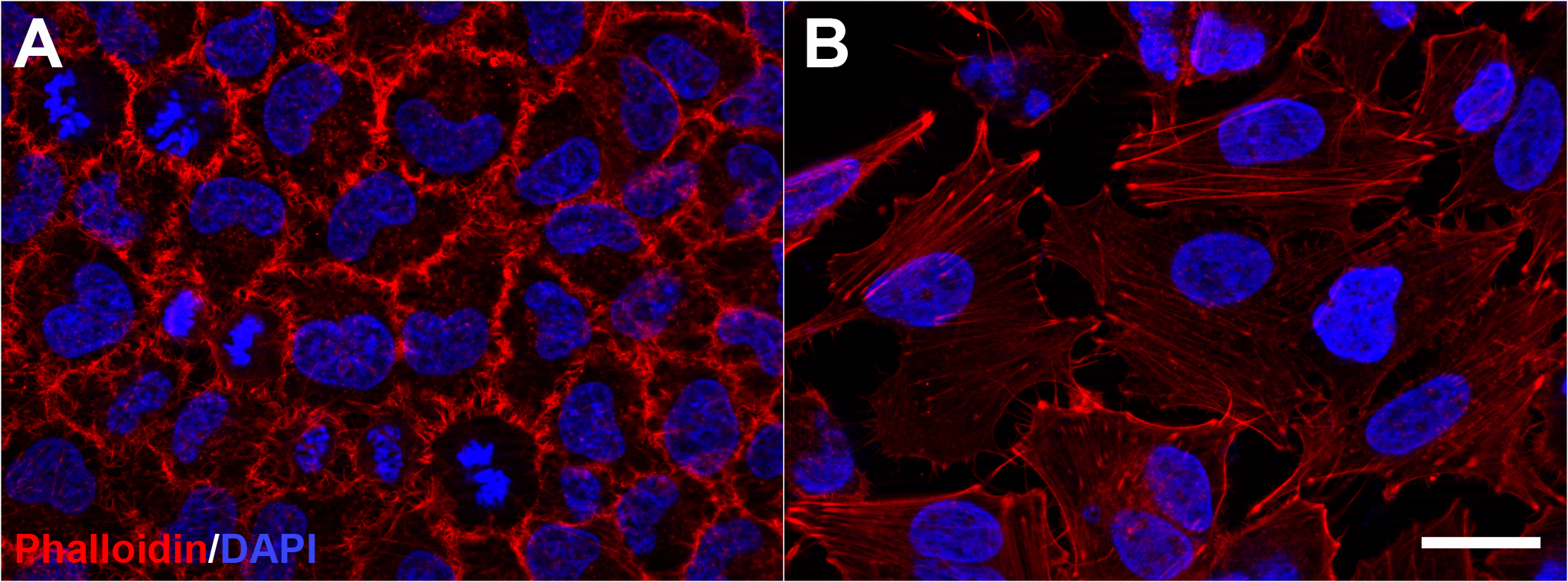

Supplement: Figure S1 — Sodium butyrate induces F-actin rearrangements in H460 cells. H460 cells were gown in the absence (A) or presence (B) of 10 mM NaB. Cells were labeled with rhodamine-conjugated phaloidin (red) and DAPI (blue), to allow visualization of actin citoeskeleton and nuclei, respectively. Representative immunofluorescence images obtained using a Zeiss AxioObserver Z1 are shown. Bars: 30 µm. (TIF) [file pone.0022264.s001.tif]

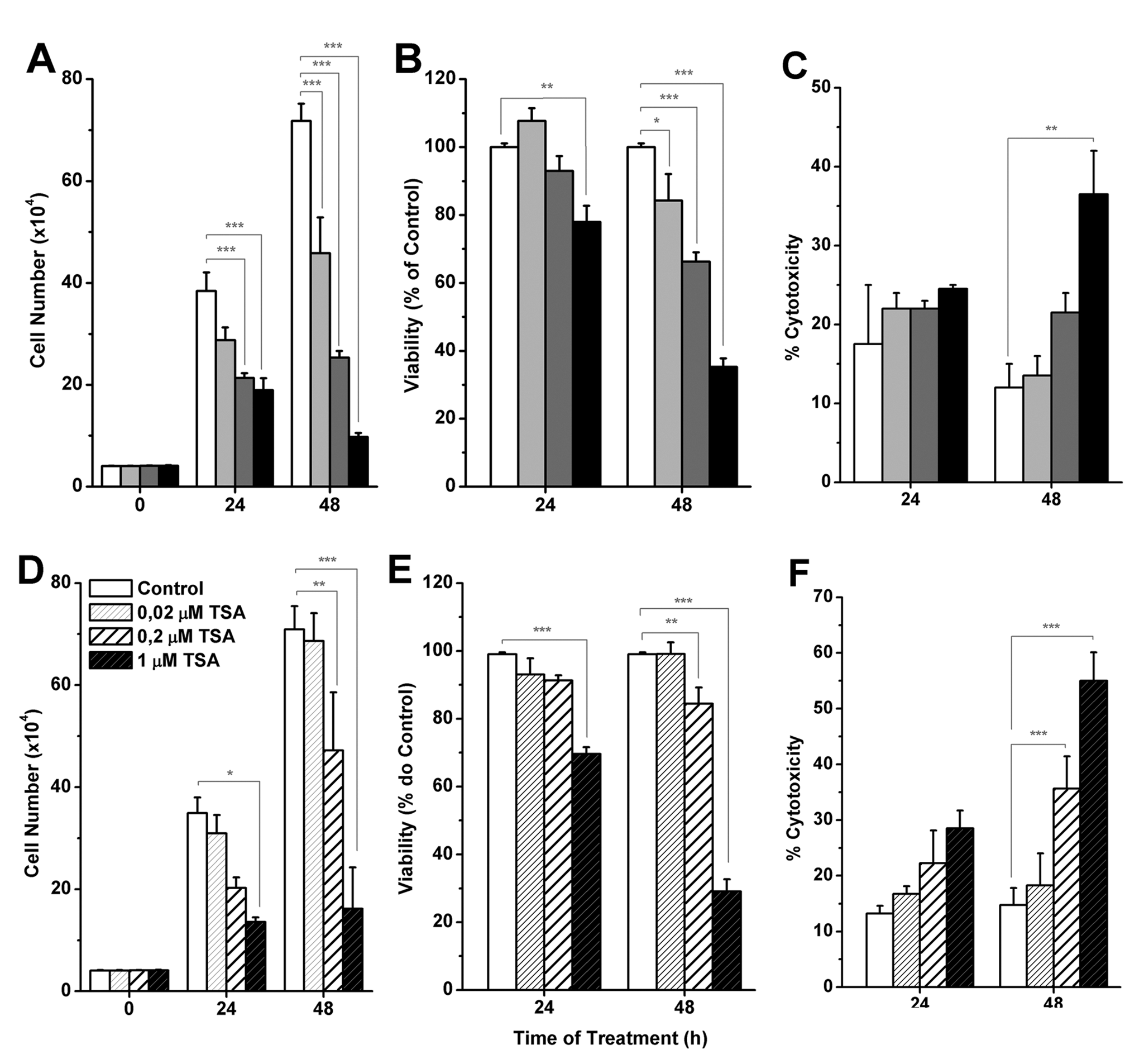

Supplement: Figure S2 — Sodium butyrate and trichostatin A induce time and dose-dependent inhibition of growth in H460 cells. Cells were grown in the absence or presence of various concentrations of NaB (1, 3 and 10 mM; A–C) or TSA (0.02, 0.2 and 1 µM; D–F) for 24 or 48 h. At indicated times, cell number and viability were analyzed by trypan blue exclusion (A and D) and MTT assay (B and E). Also, citotoxicity of the different amounts of NaB or TSA was estimated by measurement of lactate dehydrogenase (LDH) release using CytoTox96 Non-Radioactive Cytotoxicity Assay Kit (Promega) (C and F). Values represent mean ± SEM; N = 3, *P<0.05; **P<0.01; ***P<0.001. (TIF) [file pone.0022264.s002.tif]

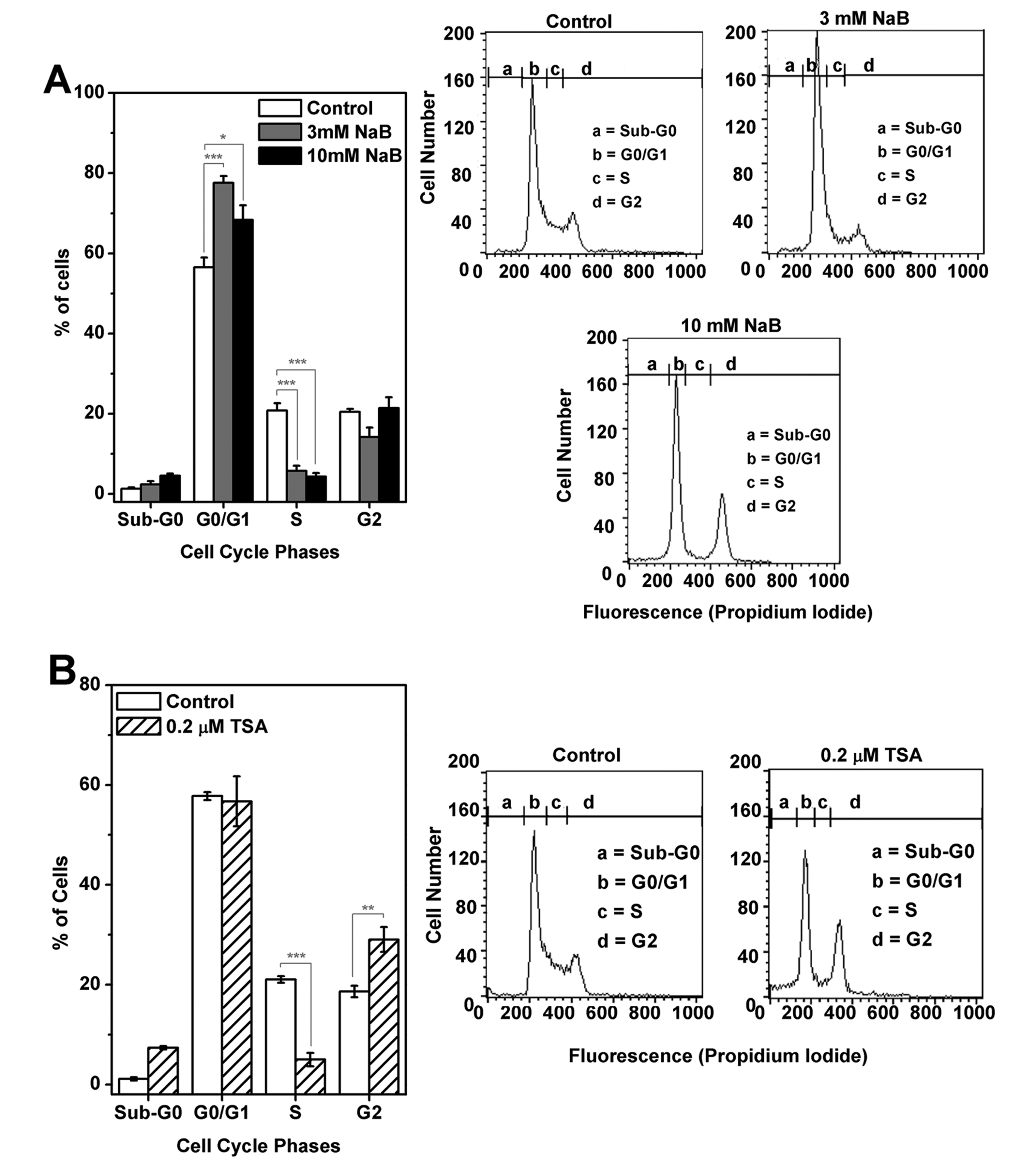

Supplement: Figure S3 — Sodium butyrate and trichostatin A induces cell cycle arrest and inhibits cellular DNA synthesis. H460 cells were treated or not with 3 or 10 mM NaB (A) and 0.2 µM TSA (B) for 24 h. Posteriorly cells were harvested, permeabilized, incubated with propidium iodide and had its DNA content analyzed by flow cytometry using FACScan. Values represent mean ± SEM; N = 4, *P<0.05; **P<0.01; ***P<0.001. (TIF) [file pone.0022264.s003.tif]

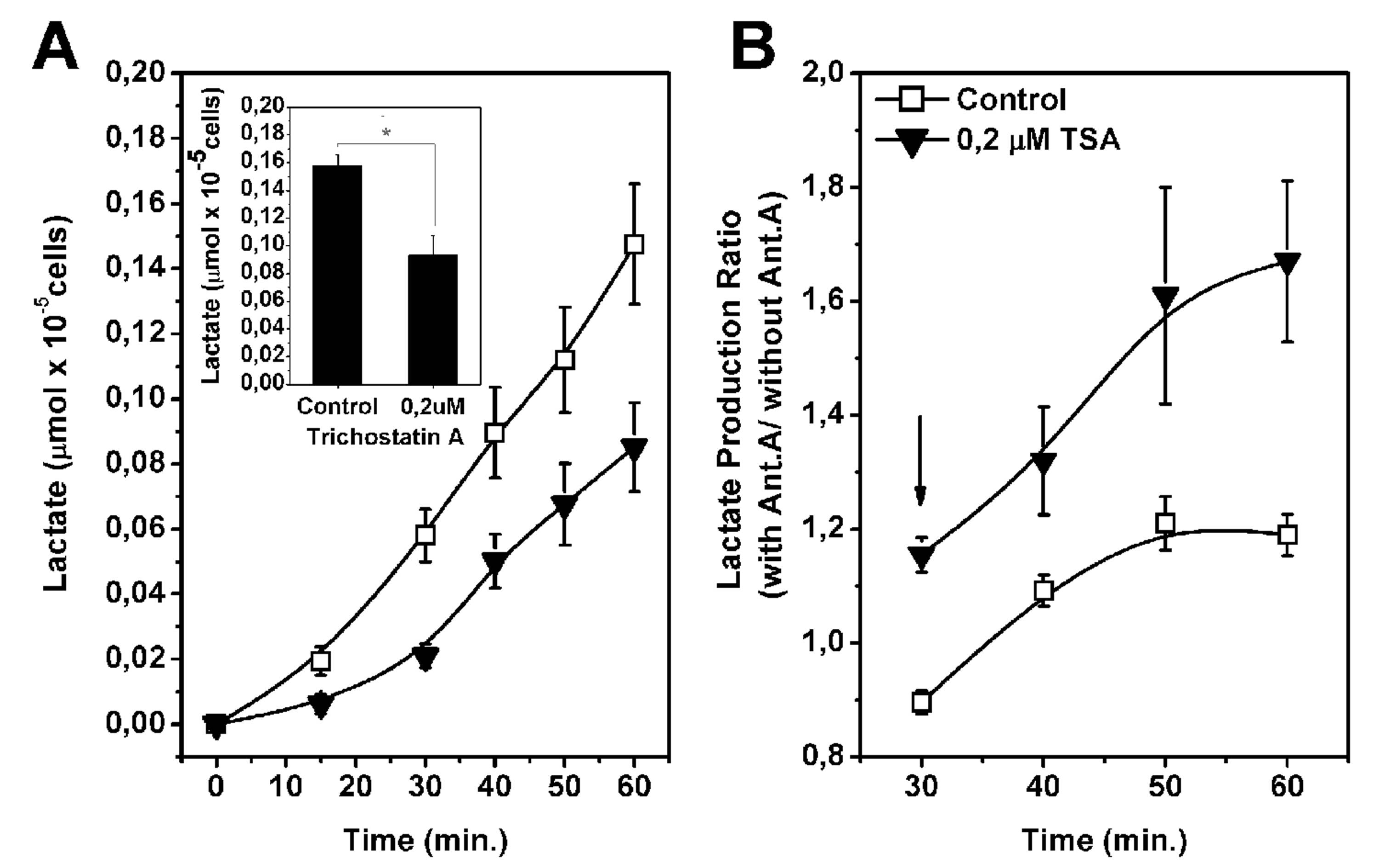

Supplement: Figure S4 — Trichostatin A reduces lactate production in H460 cells. After 24 h of treatment with 0.2 µM TSA, H460 cells were incubated with glucose-supplemented medium. Aliquots of supernatants were collected every 10 min. and incubated in hidrazine buffer pH 9.2, with an excess of NAD+ and lactate dehydrogenase (LDH) for measurement of lactate release. (A) Kinetics of lactate release and representation of lactate release after 60 minutes (inset). (B) After 30 minutes of incubation with glucose, 2 µg/ml antimycin A was added to the culture. Aliquots of the supernatant were taken at 10 minutes intervals and lactate released was measured. The lactate production ratio of H460 cells in the presence and absence of antimycin A evidences the stimulation on lactate production when oxidative phosphorylation was inhibited by the addition of this drug (indicated by the black arrow). Values represent mean ± SEM; N = 4, *P<0.05. (TIF) [file pone.0022264.s004.tif]

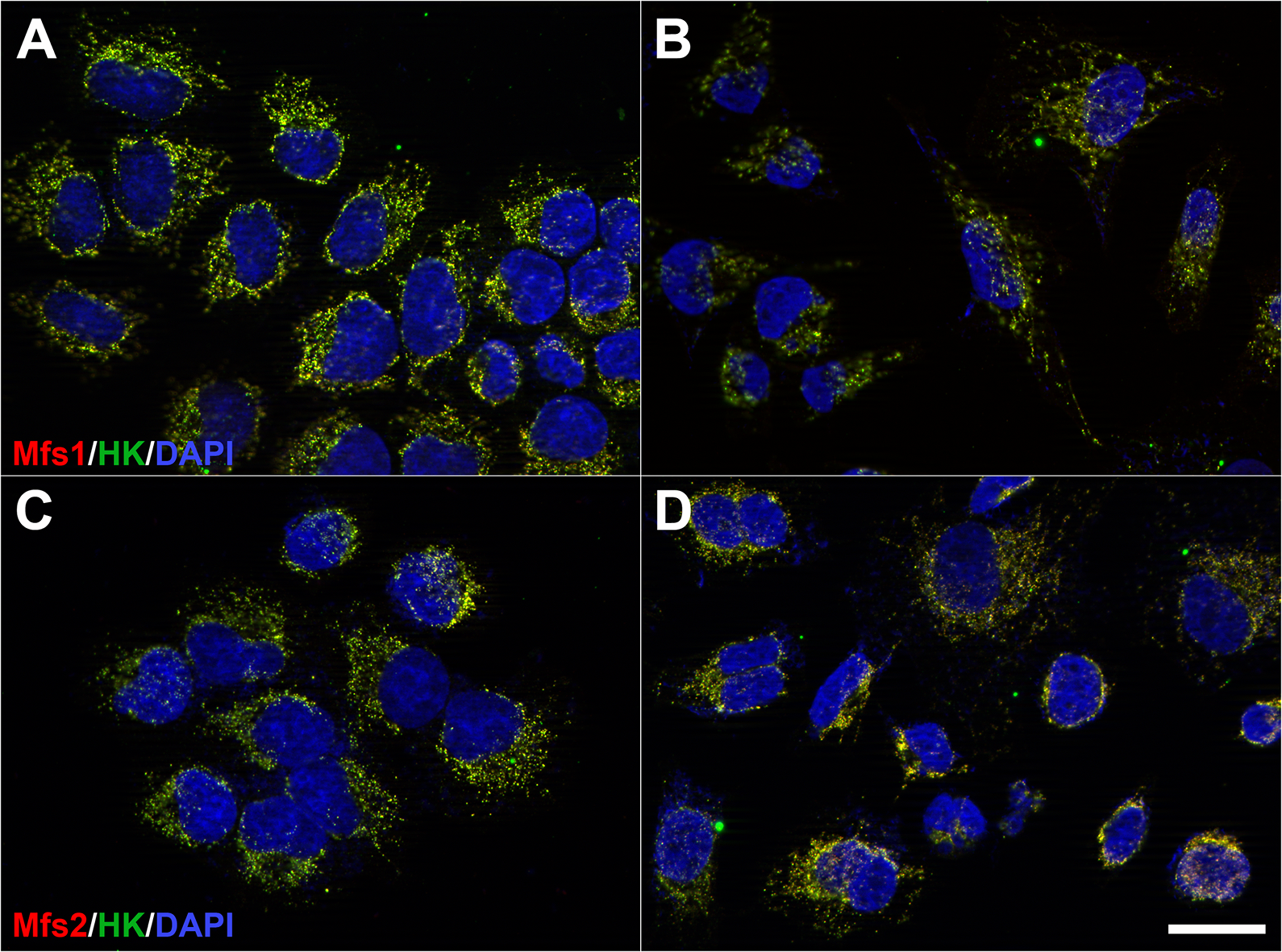

Supplement: Figure S5 — HK I is bound to mitochondria in H460 cells. Control (A and C) or NaB treated (B and D) H460 cells were labeled with antibodies against mitofusin I (A–B) or mitofusin II (C–D) (red), Hexokinase I (green). Merge HK + Mfn (yellow) and DAPI (Blue). Representative images taken with an inverted fluorescence microscope Zeiss AxioObserver Z1 are shown. Bars: 30 µm. (TIF) [file pone.0022264.s005.tif]

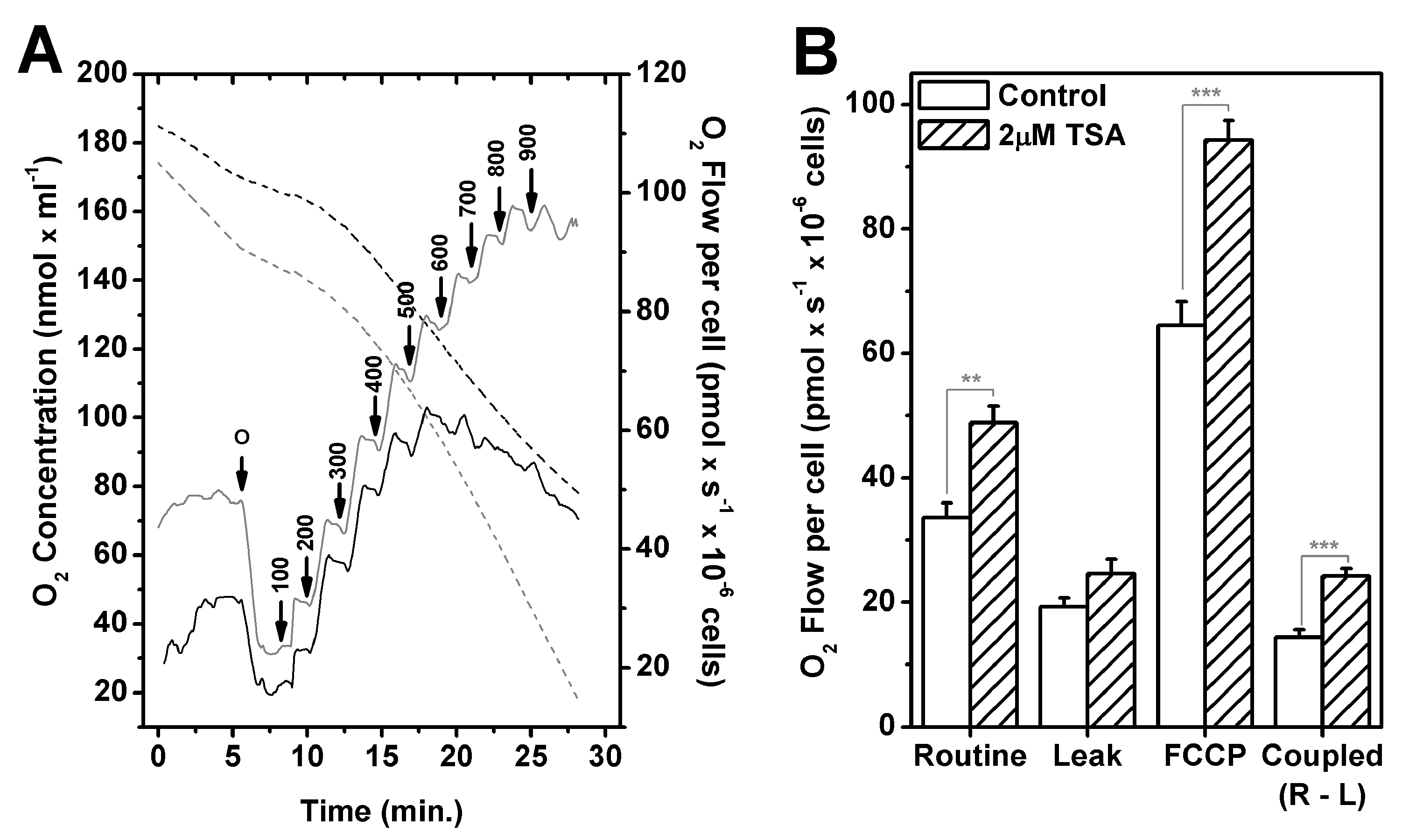

Supplement: Figure S6 — High-resolution respirometry shows an increase in oxidative metabolism subsequent to treatment with trichostatin A. (A) Representative record of oxygen concentration and flow of intact H460 cells treated or not with 0.2 µM TSA for 24 h. During the assay, cells were maintained in RPMI medium with glucose and without FBS. Black dashed line represents oxygen concentration in control cells and gray dashed line in TSA treated ones. Black solid line represents oxygen flow in control cells and gray solid line in TSA treated ones. “O” 1 µg/mL oligomycin; “arrows” indicate the titration of FCCP (nM). (B) Effect of TSA treatment on respiratory parameters of intact H460 cells. Routine respiration - basal respiration of H460 intact cells; Leak respiration - rate of oxygen consumption after the addition of oligomycin, that is, respiration not coupled to ATP synthesis; FCCP - maximum respiratory capacity (induced by the addition of FCCP); Coupled respiration - respiration coupled to ATP synthesis, obtained by subtraction of Leak from Routine respiration. During the assay, cells were maintained in RPMI medium with glucose and without FBS. Values represent mean ± SEM; N = 5, *P<0.05, **P<0.01; ***P<0.001. (TIF) [file pone.0022264.s006.tif]
