# Supplementary material for: Energy Metabolism in H460 Lung Cancer Cells: Effects of Histone Deacetylase Inhibitors
Source: PLoS One. 2011 Jul 18;6(7):e22264. doi: 10.1371/journal.pone.0022264 (PMC3138778; doi:10.1371/journal.pone.0022264)
Supplement: Table S1 — List of primer sequences used for quantitative Real Time PCR. (DOC) [file pone.0022264.s007.doc]

Table S1**.** **Primer sequences used for quantitative Real Time PCR.**

| **Actin** | Forward 5’- TTCCTTCCTGGGCATGGAGTC -3' |
| --- | --- |
|  | Reverse 5’- AGACAGCACTGTGTTGGCGTA -3' |
| **GLUT - 1** | Forward 5’- AATGCTGATGATGAACCTGCT -3’ |
|  | Reverse 5’- CAGTACACACCGATGATGAAG -3’ |
| **GLUT - 3** | Forward 5’- CTTTCTCATCCCACGCACTC -3’ |
|  | Reverse 5’- CACTCGGTCTCTCCTAAGCA -3’ |
| **Hexokinase I** | Forward 5’- GATCATCGGCACTGGCACCAA -3' |
|  | Reverse 5’- CCAAAGGCTCCCCATTCTGTA -3' |
| **Hexokinase II** | Forward 5’- ATGAGGGGCGGATGTGTATCA -3' |
|  | Reverse 5’- GGTTCAGTGAGCCCATGTCAA -3' |
| **Mitofusin I** | Forward 5’- AGCAAAAAGCACAGGGGATG -3' |
|  | Reverse 5’- GCTGACTGCGAGATACACT -3' |
| **NADH dehydrogenase** | Forward 5’- TCAAACTACGCCCTGATCGG -3' |
|  | Reverse 5’- GGAGAGGTTAAAGGAGCCACT -3' |
