# Supplementary material for: Energy Metabolism in H460 Lung Cancer Cells: Effects of Histone Deacetylase Inhibitors
Source: PLoS One. 2011 Jul 18;6(7):e22264. doi: 10.1371/journal.pone.0022264 (PMC3138778; doi:10.1371/journal.pone.0022264)
Supplement: Table S2 — Effect of sodium butyrate on ATP content in H460 cells. ATP content was assayed using an enzymatic method with hexokinase and glucose-6-phosphate dehydrogenase [40]. Values represent mean ± SEM; N = 3. (DOC) [file pone.0022264.s008.doc]

TABLE S2. **Effect of sodium butyrate on ATP content in H460 cells.**

| **ATP content (nmol x 10^-7^)** | |
| --- | --- |
| **Control**  1.85±0.5 | **NaB**  2.8±0.2 |
