# Supplementary material for: Energy Metabolism in H460 Lung Cancer Cells: Effects of Histone Deacetylase Inhibitors
Source: PLoS One. 2011 Jul 18;6(7):e22264. doi: 10.1371/journal.pone.0022264 (PMC3138778; doi:10.1371/journal.pone.0022264)
Supplement: Method S1 — ATP Content. (DOC) [file pone.0022264.s009.doc]

**Supporting method information (Method S1)**

*ATP Content -* Cells were cultured in the absence or presence of 10 mM NaB for 24 h. After treatment cells were disrupted with liquid N2 and cellular proteins were precipitated with trichloroacetic acid (6%). The samples were neutralized with 1M Tris base. ATP measurement was performed in a buffer, containing 20 mM Tris-Cl pH 7.4, 5 mM MgCl_2_, 10 mM glucose, 0,5 mM , 10 mM glucose, 0,5 mM β-NAD^+^, 1 unit/mL G6PDH and 10 units/mL hexocinase (yeast). The reaction was started by the addition of extract and carried out for 15 min. The absorbance due to formation of NADH was monitored in a microplate reader (SpectraMax M5, Molecular Devices) at 340 nm and was correlated with the presence of ATP on samples from a standard curve.
