# Supplementary material for: Energy Metabolism in H460 Lung Cancer Cells: Effects of Histone Deacetylase Inhibitors
Source: PLoS One. 2011 Jul 18;6(7):e22264. doi: 10.1371/journal.pone.0022264 (PMC3138778; doi:10.1371/journal.pone.0022264)
Supplement: Method S2 — Immunocytochemistry. (DOC) [file pone.0022264.s010.doc]

**Supporting method information (Method S2)**

*Immunocytochemistry* - Cells were cultured in the absence or presence of 10 mM NaB for 24 h. After treatment, cells were fixed with 4% paraformaldehyde in 0.1 M PBS and immunolabeled with antibodies anti-mitofusin I, anti-mitofusin II (Santa Cruz; 1:1000 dilution) or anti-Hexokinase I (Abcam; 1:1000 dilution) and then incubated for 3 h at 23°C with anti-goat Alexa Fluor 488 or anti-mouse Alexa Fluor 594 - conjugated secondary antibody, respectively (1:1000 dilution; Molecular Probes). Coverslips were mounted using Prolong Gold (Molecular Probes). Actin filaments were labeled using Alexa 488-conjugated phalloidin for 15 min. at 23°C, according to manufacturer's instructions. Nuclei were labeled using DAPI for 5 min. at 23°C. Images were analyzed using a Zeiss Axio Observer.Z1 inverted fluorescence microscope in a 60X objective and AxioCam MRm digital camera. Analysis of immunofluorescence data was carried out using NIH Image J.
